# Supplementary material for: Multidrug-resistant non-typhoidal Salmonella enterica from chickens, farmworkers, and environments: One health implications from Northwestern Ethiopia
Source: PLoS One. 2025 Oct 8;20(10):e0333591. doi: 10.1371/journal.pone.0333591 (PMC12507302; doi:10.1371/journal.pone.0333591)
Supplement: S1 Fig — (PDF) [file pone.0333591.s001.pdf]

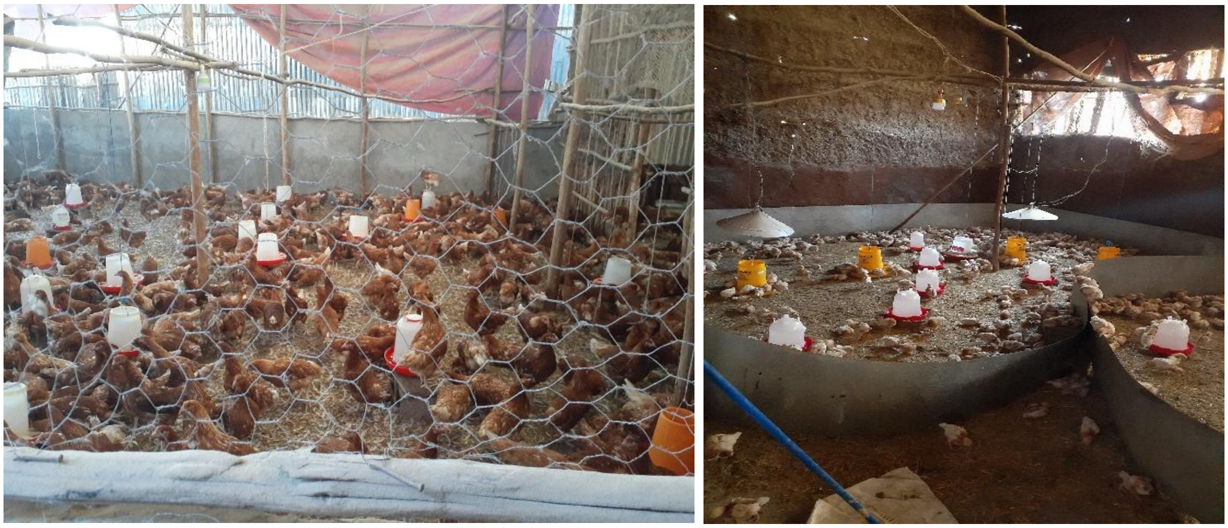

Poultry Farms

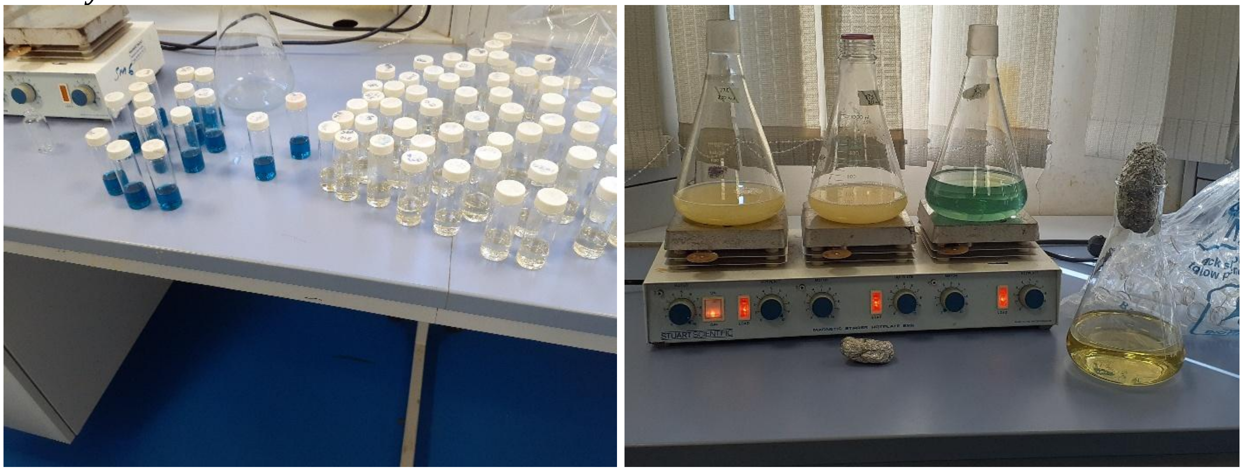

Culture Media preparations

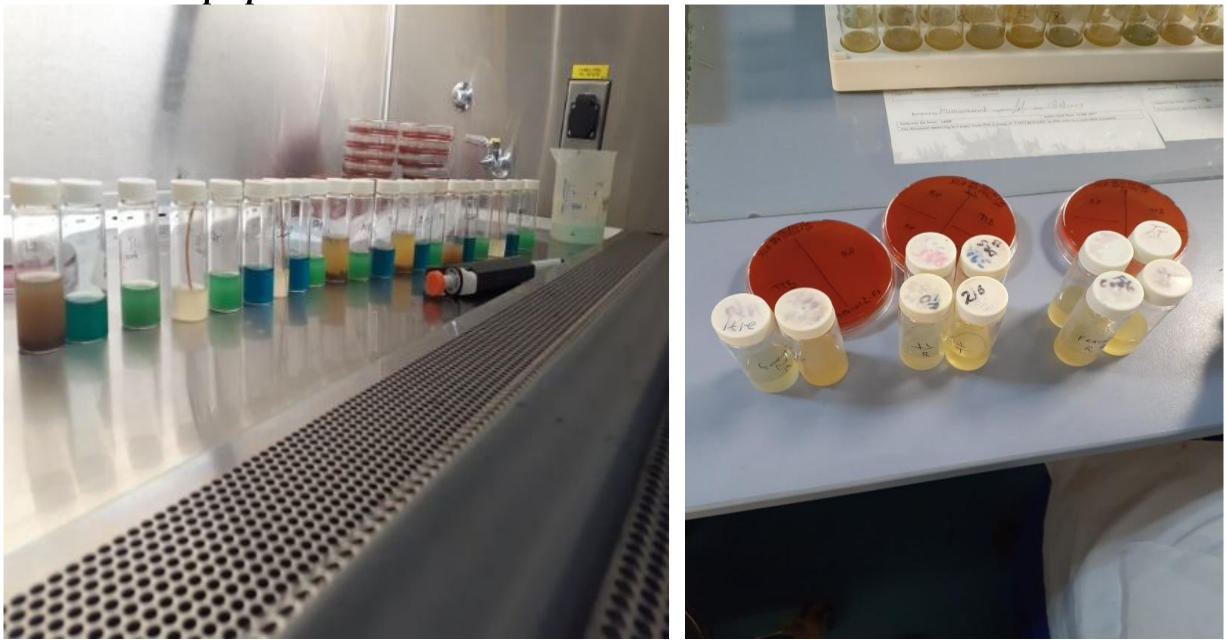

Enrichment and isolation procedures

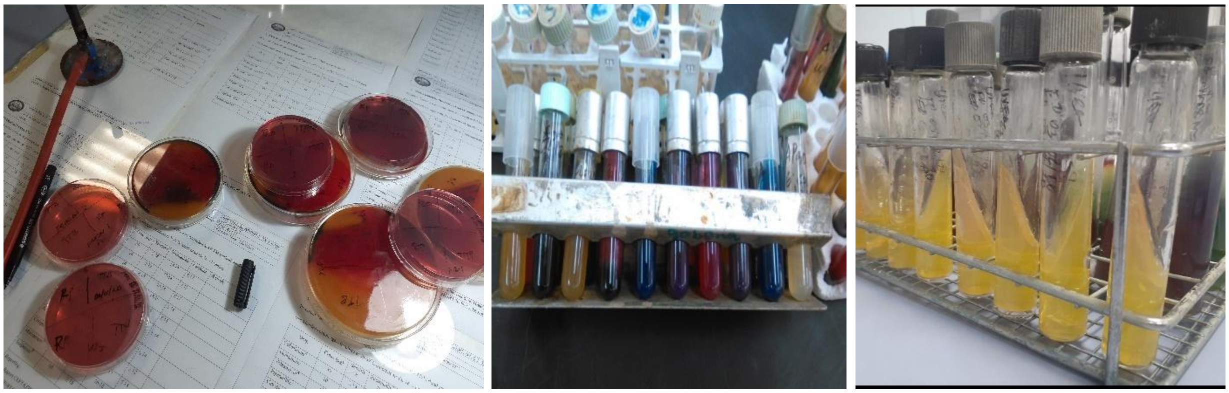

Colonies of Salmonella sp. on XLD agar, and Biochemical tests

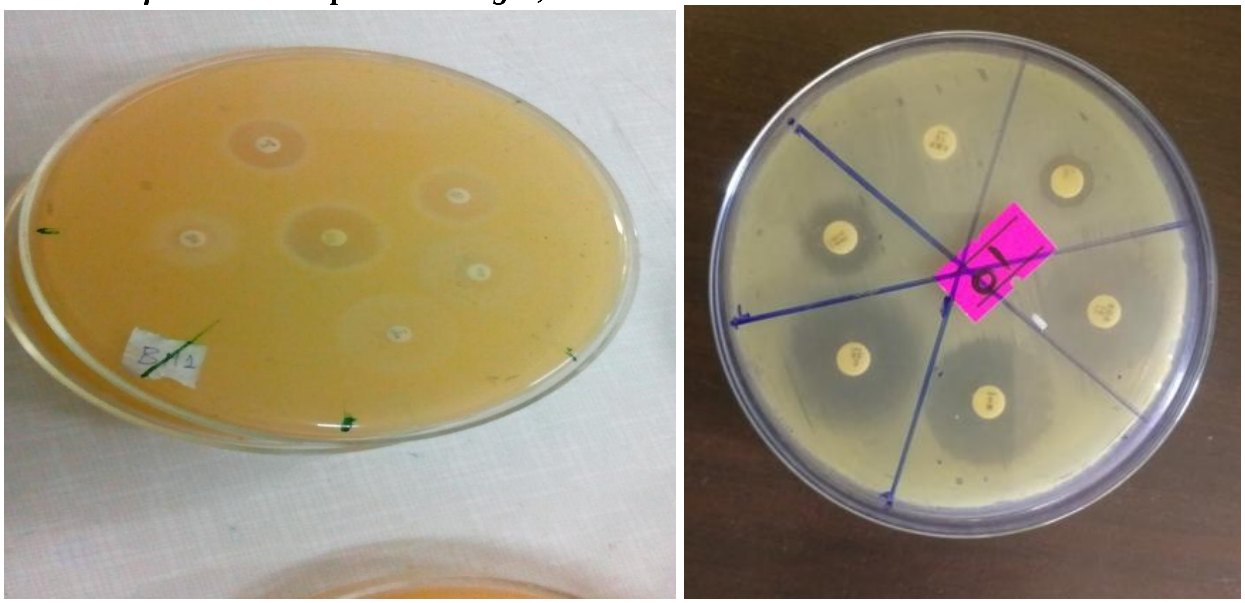

Antimicrobial susceptibility testing

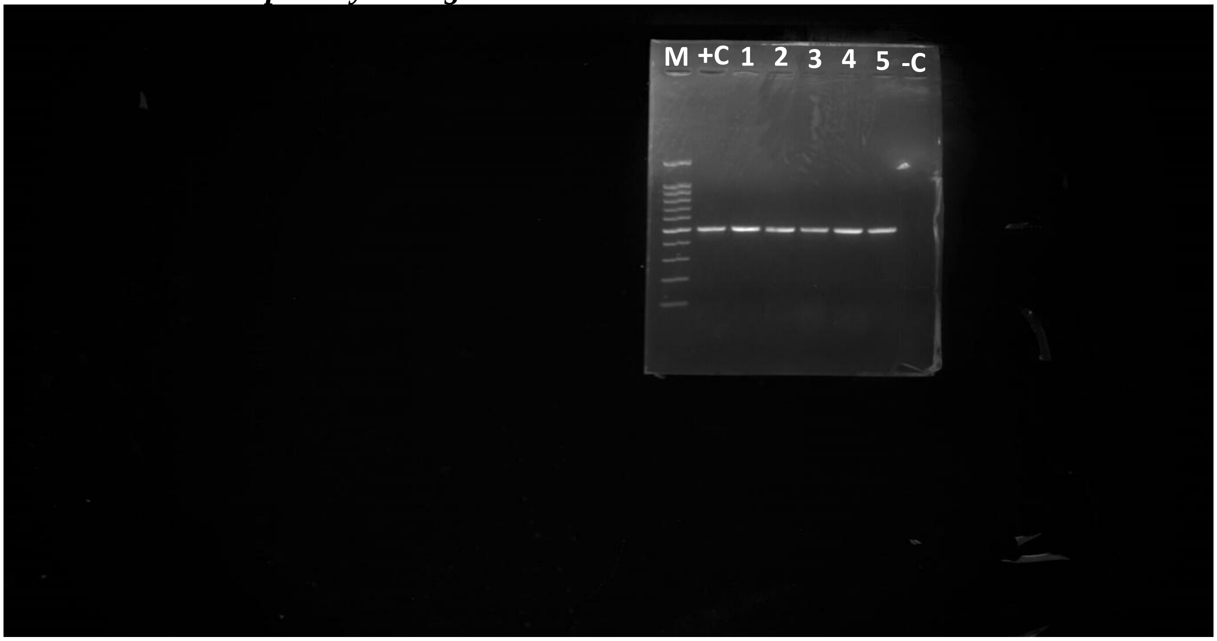

Genus *Salmonella* (496 bp) M = molecular marker; +C = positive control; 1-5 = samples; -C = negative control

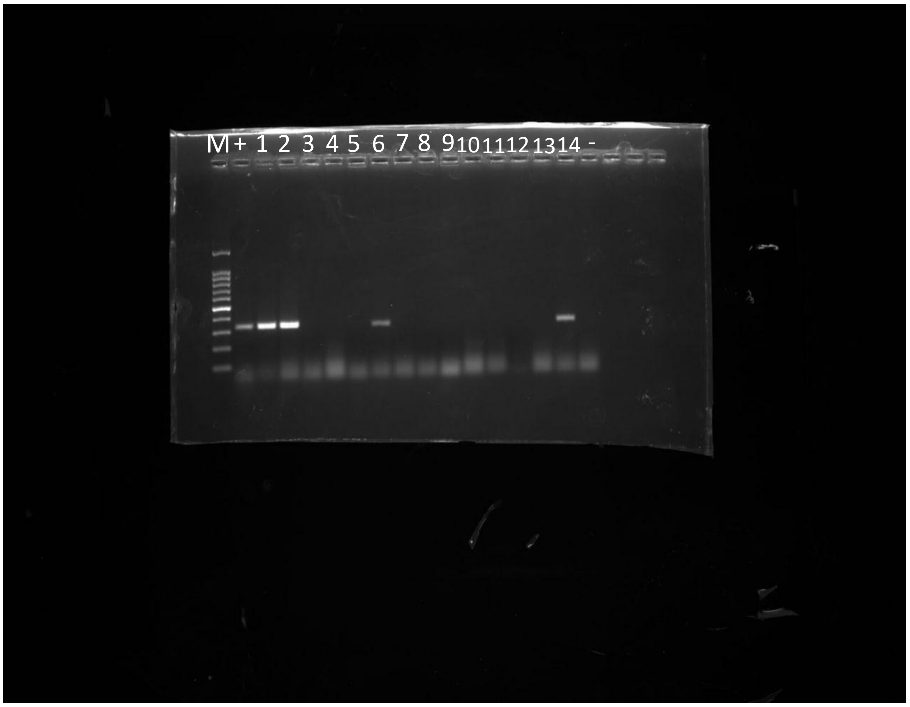

***Salmonella* Enteritidis (304 bp)** M = molecular marker; + = positive control; 1-14 = samples; - = negative control

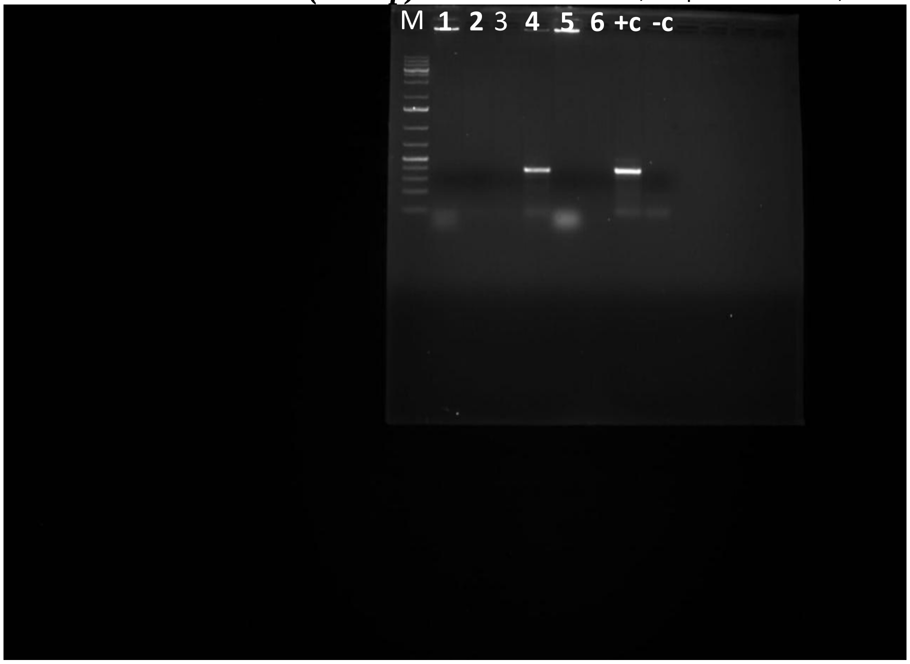

***Salmonella* Typhimurium (401 bp)** M = molecular marker; 1-6 = samples; +C = positive control; -C = negative control

S1 raw images. Representative raw images collected during the study period, including sample collection, *Salmonella* isolation, biochemical and molecular confirmation, serotype identification, and antimicrobial susceptibility testing.
