# Supplementary material for: Multidrug-resistant non-typhoidal Salmonella enterica from chickens, farmworkers, and environments: One health implications from Northwestern Ethiopia
Source: PLoS One. 2025 Oct 8;20(10):e0333591. doi: 10.1371/journal.pone.0333591 (PMC12507302; doi:10.1371/journal.pone.0333591)
Supplement: S2 File — (PDF) [file pone.0333591.s002.pdf]

S1 File: Semi-structured questionnaires used to gather information on farm size, bird type, bird age, history of antimicrobial use, and other poultry farm management practices from 22 poultry farms.

Date: ____________

*Code number*__________________________

1. Region_______________District/Woreda_______________KebeleVillage____________
   Farm name______________ Geo reference ___________
2. Owners name: ____________________________Gender_______ Age_____
   Years of farming experience: ______________
3. Type of Farm: ☐ Broiler ☐ Layer
4. Scale of Farm: ☐ Large ☐ Small ☐ Backyard/ scavenging
   ☐ Family ☐ Enterprise ☐ Private
5. Location of farm: ☐ Urban ☐ Peri-Urban ☐ Rural
6. How many birds do you have on your farm? ____________.
7. What are the names of the chicken breeds you raise? _______________.
8. Please specify the age of the batch of chickens. _______________________.
9. What are the sources of foundation or replacement stock?
   ☐ Purchase ☐ Inherited ☐ Hatched ☐ Other, specify-------------
10. What type of housing system do you use for your poultry raising?
    ☐ Cage ☐ Litter/Floor ☐ Slatted floor system
11. If your answer to Q10 is a floor system, please specify the litter materials___________.
12. What type of design was used for constructing the poultry house?
    ☐ Conventional/ open-sided ☐ Controlled Environment
13. Please indicate the types of house ventilation you use in your poultry farm.
    ☐ Natural air flow ☐ Mechanical air movement/fans
14. What do you feed your birds?
    ☐ Purchased formulated feed ☐ Home-made feed ☐ Both ☐ Other, specify_____
15. When do you feed your chickens?
    ☐ Morning and evening ☐ Morning and afternoon ☐ Morning, afternoon, and evening
    ☐ Free access

16. What is the source of water?

☐ Tap water ☐ Treated well water ☐ Untreated well water

17. How frequently do you provide water to your poultry?

☐ Free access ☐ Morning only ☐ Morning and evening

☐ Morning, afternoon and evening ☐ If other (specify) _______________

18. Do you experience serious disease outbreaks??

☐ Yes ☐ No. If yes, how often does it occur? ______________

19. What do you do when birds fall sick?

☐ Treat them myself ☐ Call Vet. Doctor ☐ Kill them immediately

☐ Consume them immediately ☐ Sell them immediately ☐ if other, specify--------

20. Are there sudden and high mortality rates of flocks on your farm?

☐ Yes ☐ No.

21. What are the common diseases and symptoms you have experienced in your flock?

Name of disease	Symptoms Favorable	Seasons (Local)	Treatment
1.			
2.			
3.			
4.			

22. Do you use antimicrobial drugs on the farm?

☐ Yes ☐ No.

23. If the response is yes to Q21, choose the purpose of use.

☐ Treatment ☐ Prophylaxis ☐ As a feed additive

24. What are the common antibiotic drugs used on the farm? _________________

25. Do you practice annual vaccination of your chicken?

☐ Yes ☐ No

26. If your answer to Q25 is “Yes”, against which diseases are your chickens vaccinated? (Please tick all that apply)

☐ Newcastle diseases ☐ Marek’s Disease ☐ Fowl typhoid ☐ Gumboro (infectious bursa disease) ☐ Infectious bronchitis

27. Do you have dedicated personal protective equipment (PPE) for the poultry house workers?

☐ Yes ☐ No

28. Are foot dips available at the entry of each poultry house?

☐ Yes ☐ No

29. Do you have a cleaning and disinfection program for the houses and poultry equipment?

☐ Yes ☐ No

30. Do you experience the presence of rodents and pests on your farm?

☐ Yes ☐ No

31. Are other animals keeping on your farm?

☐ Yes ☐ No

32. If the answer to Q30 is yes, please specify (please tick all that apply)

☐ Sheep ☐ Cattle ☐ Dog/Cat ☐ Horse
