# Supplementary material for: Multidrug-resistant non-typhoidal Salmonella enterica from chickens, farmworkers, and environments: One health implications from Northwestern Ethiopia
Source: PLoS One. 2025 Oct 8;20(10):e0333591. doi: 10.1371/journal.pone.0333591 (PMC12507302; doi:10.1371/journal.pone.0333591)
Supplement: Attachment — Submitted filename: PONE-D-25-25886.pdf [file pone.0333591.s003.pdf]

PLOS ONE

Multidrug-resistant non-typhoidal Salmonella enterica from chickens, farmworkers, and environments in Northwestern Ethiopia: One Health Implications

--Manuscript Draft--

**Manuscript Number:**	PONE-D-25-25886
**Article Type:**	Research Article
**Full Title:**	Multidrug-resistant non-typhoidal Salmonella enterica from chickens, farmworkers, and environments in Northwestern Ethiopia: One Health Implications
**Short Title:**	Multidrug-resistant non-typhoidal Salmonella enterica in Northwestern Ethiopia
**Corresponding Author:**	Mequanint Addisu Belete<br>Debre Markos University<br>Debre Markos, ETHIOPIA
**Keywords:**	Antimicrobial resistance; Nontyphoidal Salmonella; occurrence; One Health; risk factors
**Abstract:**	Background<br><br>Multidrug-resistant Salmonella Enteritidis and Salmonella Typhimurium are among the most significant foodborne zoonotic pathogens, particularly associated with poultry, a major global public health concern. Despite their significance, limited data are available on the epidemiology of nontyphoidal Salmonella in poultry production systems in Ethiopia. This cross-sectional study was conducted to estimate the prevalence, identify risk factors, and evaluate the antimicrobial resistance profiles of Salmonella enterica isolates from poultry farms in northwestern Ethiopia.<br><br>Methods<br><br>Standard bacteriological methods, PCR-based detection, and serotyping were employed to investigate the presence of Salmonella in chicken (n = 126), environmental (n = 198), and human (n = 45) samples collected from 22 poultry farms. Antimicrobial susceptibility profiles were determined using the Kirby–Bauer disk diffusion method. Data from questionnaires and Chi-square statistics analysis were used to identify risk factors associated with the occurrence of Salmonellosis.<br><br>Results<br><br>Nontyphoidal Salmonella species were detected on 18.1% (4/22) of the farms. Salmonella enterica was recovered from 3.1% (6/198) of environmental boot samples, 3.2% (4/126) of cloacal swabs, and 4.4% (2/45) of human stool samples. Two Salmonella serotypes were identified from a total of 12 Salmonella isolates: S. Enteritidis (41.6%, 5/12) and S. Typhimurium (16.6%, 2/12). All Salmonella isolates demonstrated complete resistance to ampicillin (100%) and tetracycline (100%) and exhibited multiple drug resistance patterns, with a high multiple antibiotic resistance index ranging from 0.45 to 0.55. The prevalence of Salmonella was significantly associated with larger flock sizes ($p = 0.044$), the absence of foot baths ($p = 0.003$), and the presence of other animal species on the farm ($p = 0.002$).<br><br>Conclusion<br><br>This study provides valuable insights into the occurrence of predominant multidrug-resistant nontyphoidal Salmonella serotypes on commercial poultry farms in northwestern Ethiopia and identifies key factors contributing to antimicrobial resistance and nontyphoidal salmonellosis infections. Therefore, implementing enhanced intervention strategies and conducting ongoing large-scale One Health genomic surveillance are crucial to accurately identify diverse sources and transmission dynamics of Salmonella infection and effectively control the spread of antimicrobial resistance.
**Order of Authors:**	Azeb Bayu Mengistu

Powered by Editorial Manager® and ProduXion Manager® from Aries Systems Corporation

	Mequanint Addisu Belete
	Habtamu Tassew
	Haregua Yesigat Kassa
	Beyenech Gebeyehu Alemu
	Hailehizeb Cheru Tegegne
	Demelash Areda
**Additional Information:**	
**Question**	**Response**
**Financial Disclosure**<br><br>Enter a financial disclosure statement that describes the sources of funding for the work included in this submission. Review the submission guidelines for detailed requirements. View published research articles from *PLOS ONE* for specific examples.<br><br>This statement is required for submission and **will appear in the published article** if the submission is accepted. Please make sure it is accurate.<br><br>**Funded studies**<br>Enter a statement with the following details:<br>• Initials of the authors who received each award<br>• Grant numbers awarded to each author<br>• The full name of each funder<br>• URL of each funder website<br>• Did the sponsors or funders play any role in the study design, data collection and analysis, decision to publish, or preparation of the manuscript?<br><br>Did you receive funding for this work?	The author(s) received no specific funding for this work.
**Competing Interests**<br><br>Use the instructions below to enter a competing interest statement for this submission. On behalf of all authors, disclose any competing interests that could be perceived to bias this work—acknowledging all financial support and any other relevant financial or non-financial competing interests.	The authors have declared that no competing interests exist.

This statement is required for submission and **will appear in the published article** if the submission is accepted. Please make sure it is accurate and that any funding sources listed in your Funding Information later in the submission form are also declared in your Financial Disclosure statement.<br><br>View published research articles from *PLOS ONE* for specific examples.<br><br>**NO authors have competing interests**<br><br>Enter: *The authors have declared that no competing interests exist.*<br><br>**Authors with competing interests**<br><br>Enter competing interest details beginning with this statement:<br><br>*I have read the journal's policy and the authors of this manuscript have the following competing interests: [insert competing interests here]*<br><br>* typeset	
**Ethics Statement**<br><br>Enter an ethics statement for this submission. This statement is required if the study involved:<br><br>• Human participants<br>• Human specimens or tissue<br>• Vertebrate animals or cephalopods<br>• Vertebrate embryos or tissues<br>• Field research<br><br>Write "N/A" if the submission does not require an ethics statement.<br><br>General guidance is provided below. Consult the submission guidelines for detailed instructions. **Make sure that all**	The study protocol (Ref. No: BU/ECRC/1/112/13/2020) was reviewed and approved by the institutional Ethical and Environmental Considerations Review Committee of Bahir Dar University. Both oral and written Informed consent were obtained from study participants after a thorough explanation of the purpose of the study.

Powered by Editorial Manager® and ProduXion Manager® from Aries Systems Corporation

information entered here is included in the Methods section of the manuscript.<br><br>**Format for specific study types**<br><br>**Human Subject Research (involving human participants and/or tissue)**<br>• Give the name of the institutional review board or ethics committee that approved the study<br>• Include the approval number and/or a statement indicating approval of this research<br>• Indicate the form of consent obtained (written/oral) or the reason that consent was not obtained (e.g. the data were analyzed anonymously)<br><br>**Animal Research (involving vertebrate animals, embryos or tissues)**<br>• Provide the name of the Institutional Animal Care and Use Committee (IACUC) or other relevant ethics board that reviewed the study protocol, and indicate whether they approved this research or granted a formal waiver of ethical approval<br>• Include an approval number if one was obtained<br>• If the study involved *non-human primates*, add *additional details* about animal welfare and steps taken to ameliorate suffering<br>• If anesthesia, euthanasia, or any kind of animal sacrifice is part of the study, include briefly which substances and/or methods were applied<br><br>**Field Research**<br><br>Include the following details if this study involves the collection of plant, animal, or other materials from a natural setting:<br>• Field permit number<br>• Name of the institution or relevant body that granted permission	
**Data Availability**<br><br>Authors are required to make all data underlying the findings described fully available, without restriction, and from the time of publication. PLOS allows rare exceptions to address legal and ethical	Yes - all data are fully available without restriction

concerns. See the PLOS Data Policy and FAQ for detailed information.<br><br>A Data Availability Statement describing where the data can be found is required at submission. Your answers to this question constitute the Data Availability Statement and **will be published in the article**, if accepted.<br><br>**Important:** Stating 'data available on request from the author' is not sufficient. If your data are only available upon request, select 'No' for the first question and explain your exceptional situation in the text box.<br><br>Do the authors confirm that all data underlying the findings described in their manuscript are fully available without restriction?	
**Describe where the data may be found in full sentences. If you are copying our sample text, replace any instances of XXX with the appropriate details.**<br><br>• If the data are **held or will be held in a public repository**, include URLs, accession numbers or DOIs. If this information will only be available after acceptance, indicate this by ticking the box below. For example: *All XXX files are available from the XXX database (accession number(s) XXX, XXX.*).<br>• If the data are all contained **within the manuscript and/or Supporting Information files**, enter the following: *All relevant data are within the manuscript and its Supporting Information files.*<br>• If neither of these applies but you are able to provide **details of access elsewhere**, with or without limitations, please do so. For example:<br><br>*Data cannot be shared publicly because of [XXX]. Data are available from the XXX Institutional Data Access / Ethics Committee (contact via XXX) for researchers who meet the criteria for access to confidential data.*	All relevant data are within the manuscript and its Supporting Information files.

The data underlying the results presented in the study are available from (include the name of the third party and contact information or URL).<br>• This text is appropriate if the data are owned by a third party and authors do not have permission to share the data.<br><br>* typeset	
Additional data availability information:	

Manuscript
Click here to access/download;Manuscript;Manuscript_Plose ONE.docx

Multidrug-resistant non-typhoidal *Salmonella enterica* from chickens, farmworkers, and environments in Northwestern Ethiopia: One Health Implications

Azeb Bayu Mengistu [1], Mequanint Addisu Belete [2,*], Habtamu Tassew [3], Haregua Yesigat Kassa [2], Beyenech Gebeyehu Alemu [3], Hailehizeb Cheru Tegegne [1], Demelash Areda[4]

[1]Department of Veterinary Science, College of Agriculture and Environmental Science, Debre Tabor University, Debre Tabor, Ethiopia

[2]Department of Veterinary Laboratory Technology, College of Agriculture and Natural Resources, Debre Markos University, Debre Markos, Ethiopia

[3]School of Veterinary Science, College of Agriculture and Environmental Sciences, Bahir Dar University, Bahir Dar, Ethiopia

[4]School of Arts and Sciences, Ottawa University, Civic Center Plaza, Surprise, Arizona, United States of America.

*Correspondence:

E-mail: mequanentaddisu@gmail.com or mequanint_addisu@dmu.edu.et (MAB)

Abstract

Background

Multidrug-resistant *Salmonella* Enteritidis and *Salmonella* Typhimurium are among the most significant foodborne zoonotic pathogens, particularly associated with poultry, ~~a major global public health concern~~. Despite their significance, limited data are available on the epidemiology of nontyphoidal *Salmonella* in poultry production systems in Ethiopia. This cross-sectional study was conducted to estimate the prevalence, identify risk factors, and evaluate the antimicrobial resistance profiles of *Salmonella enterica* isolates from poultry farms in northwestern Ethiopia.

Methods

Standard bacteriological methods, PCR-based detection, and serotyping were employed to investigate the presence of *Salmonella* in chicken (n = 126), environmental (n = 198), and human (n = 45) samples collected from 22 poultry farms. Antimicrobial susceptibility profiles were determined using the Kirby–Bauer disk diffusion method. Data from questionnaires and Chi-square statistics analysis were used to identify risk factors associated with the occurrence of Salmonellosis.

Results

Nontyphoidal Salmonella species were detected on 18.1% (4/22) of the farms. *Salmonella enterica* was recovered from 3.1% (6/198) of environmental boot samples, 3.2% (4/126) of cloacal swabs, and 4.4% (2/45) of human stool samples. Two *Salmonella* serotypes were identified from a total of 12 *Salmonella* isolates: *S.* Enteritidis (41.6%, 5/12) and *S.* Typhimurium (16.6%, 2/12). All *Salmonella* isolates demonstrated complete resistance to ampicillin (100%) and tetracycline (100%) and exhibited multiple drug resistance patterns, with a high multiple antibiotic resistance index ranging from 0.45 to 0.55. The prevalence of *Salmonella* was significantly associated with larger flock sizes (p = 0.044), the absence of foot baths (p = 0.003), and the presence of other animal species on the farm (p = 0.002).

Conclusion

This study provides valuable insights into the occurrence of predominant multidrug-resistant nontyphoidal Salmonella serotypes on commercial poultry farms in northwestern Ethiopia and identifies key factors contributing to antimicrobial resistance and nontyphoidal salmonellosis infections. Therefore, implementing enhanced intervention strategies and conducting ongoing large-scale One

Health genomic surveillance are crucial to accurately identify diverse sources and transmission dynamics of *Salmonella* infection and effectively control the spread of antimicrobial resistance.

Keywords: Antimicrobial resistance, Nontyphoidal *Salmonella*, occurrence, One Health, Risk factors

Introduction

Salmonella is a significant pathogen responsible for numerous foodborne illnesses and zoonotic diseases. Among *Salmonella enterica* subspecies *enterica*, various serovars are the primary cause of self-limiting diarrheal illnesses in both humans and animals (1,2). Globally, an estimated 93.8 million cases of nontyphoidal *Salmonella* (NTS) enteric illnesses occur annually, resulting in approximately 155,000 deaths. In contrast, Africa reported a lower estimated burden, with 2.4 million cases and 4100 deaths from NTS gastroenteritis across the World Health Organization (WHO) subregions (3). However, in Sub-Saharan Africa, NTS frequently causes invasive diseases (iNTS) characterized by severe bacteremia and extraintestinal illness, particularly affecting children and immunocompromised adults (4). NTS is also a leading cause of invasive infections and diarrhea among children and adults within Ethiopia, with a pooled prevalence rate of 57.9% (5).

Salmonellosis is a devastating disease that causes substantial economic losses and impacts the development of the poultry industry. Among the over 2500 *Salmonella* serotypes, *Salmonella* Enteritidis (*S. Enteritidis*) and *Salmonella* Typhimurium (*S. Typhimurium*) are particularly problematic in poultry and poultry products (6). Since the 1980, documented pandemics and outbreaks have consistently underscored the crucial role of *S*. Enteritidis and *S*. Typhimurium in the overall burden of *Salmonella* infections in poultry farming systems and public health settings across North America, South America, Europe, Asia, and Africa (7,8). These two serotypes exhibit remarkable adaptability to adverse environments and can persist in diverse ecological niches outside poultry farms, including food production environments and food products (9).

Contaminated poultry meat and eggs remain the principal source of human *Salmonella* infections worldwide (10). For instance, surveillance data from the United States in 2017 revealed that 29% of *Salmonella* outbreaks and 34% of associated illnesses originated from contaminated poultry. *S.* Enteritidis was the most prevalent serotype, reported in 27 outbreaks, followed by *S.* Typhimurium serotype, reported in 14 outbreaks (11). However, recent studies indicate that direct contact with infected birds or indirect exposure to feces contaminated environments can also contribute to human *Salmonella* infections, emphasizing the critical importance of the One Health framework (12,13). In North America and West Africa, genomic surveillance of *Salmonella* species has identified strains closely related to humans, poultry, and the environment, sharing similarities in phylogenetics, core genome, pangenome, and virulence markers (14,15). In Korea, a clonal comparison of *S.* Enteritidis and *S.* Typhimurium isolates from humans and broiler chickens revealed identical genetic types, demonstrating the potential for zoonotic transmission from poultry to humans (16). Moreover, recent data in Ethiopia indicate an increasing overlap between *Salmonella* strains isolated from humans and poultry fecal droppings, highlighting the need to explore the significance of *Salmonella* isolation across various sample sources (17).

The emergence and dissemination of antimicrobial resistance (AMR) in bacteria present significant global public health challenges, impacting human, animal, and environmental health (18,19). In low-income settings, factors such as the misuse or overuse of antibiotics, limited awareness of AMR, and close contact between humans and animals contribute to the proliferation of drug-resistant bacteria. Poultry, in particular, acts as a reservoir for the dissemination of antibiotic-resistant NTS strains through the food chain (20,21). A comparative genomic analysis conducted in Mexico revealed that poultry harbors multidrug-resistant nontyphoidal *Salmonella* (MDR-NTS) with AMR genotypes highly similar to those observed in human clinical isolates (22). Similarly, in Senegal, combined antimicrobial susceptibility testing and whole genome analysis of *Salmonella* isolates from humans and chickens indicated the potential transmission of MDR serotypes from chickens to humans, identifying broilers as a source of antimicrobial resistance (23).

Numerous studies conducted in Ethiopia have documented a significant increase in the prevalence of MDR-NTS within the poultry sector. Tadesse Eguale (24) reported a high prevalence of multidrug-resistant (MDR) *S.* Saintpaul and *S.* Kentuck serotypes on poultry farms in central Ethiopia. Similarly, other studies have disclosed that 69.5% to 96.77% of *Salmonella* isolates from poultry farms in urban and peri-urban areas of central Ethiopia exhibited MDR (17,25,26). Notably, a high proportion—93.4% (42/45)—of *Salmonella* isolates from the poultry industry in southern Ethiopia were found to be MDR (27). However, the incidence and impact of these MDR pathogens remain poorly understood, primarily due to the absence of robust, coordinated laboratory and epidemiological surveillance systems (28).

Previous studies investigating the occurrence, serotype distribution, and antimicrobial susceptibility profile of NTS on poultry farms in Ethiopia have been limited in both number and geographical scope. Although the region is a major poultry producer, the characteristics and antibiotic resistance profile of NTS on poultry farms in Bahir Dar, located in northwestern Ethiopia, remain largely unexplored. Furthermore, research on non-typhoidal salmonellosis from a One Health perspective—which emphasizes the interconnectedness of animal, human, and environmental health—is scarce in this region. Therefore, this study aimed to investigate the prevalence, identify potential determinant factors, and determine the most common serotypes and antimicrobial resistance patterns of NTS isolated from poultry, humans, and farm environments in Bahir Dar City, northwestern Ethiopia.

Materials and methods

Ethical considerations

The study protocol (Ref. No: BU/ECRC/1/112/1.3/2020) was reviewed and approved by the institutional Ethical and Environmental Considerations Review Committee of Bahir Dar University. Written informed consent was obtained from study participants after a thorough explanation of the purpose of the study.

~~Description of the~~ study area

The study was conducted in Bahir Dar, the capital administrative city of Amhara National Regional State, in the northwestern part of Ethiopia. Geographically, Bahir Dar is situated between 11.29° to 11.38° north latitude and 37.23° to 37.36° east longitude, with an average elevation estimated to be 1810 m above sea level. The city has an average annual temperature of 20.85 °C and precipitation of 1419 mm (29). In recent years, commercial poultry farming has seen significant growth in urban and peri-urban areas, providing a source of income through the sale of eggs and live birds. However, the proximity of many of these farms to residential areas raises concerns about the potential transmission of pathogens and the spread of antibiotic resistance.

Study design, sampling, and study samples

A cross-sectional study was conducted within a period of 9 months (November 2020-July 2021. Study subjects included chickens across various age groups (<2 months, 2–6 months, 7–12 months, >12 months) (24) and their close human contacts on selected poultry farms. Twenty-two poultry farms were randomly selected from the list provided by the Bahir Dar Agricultural Development Office. These included 10 small farms (≤ 1000 birds) and 12 large farms (> 1000 birds). The number of live birds sampled per farm was proportionally allocated. Farms without official records and lacking contact with livestock extension services or veterinary officers were excluded from the study. Data collection involved direct personal observations and structured questionnaire surveys to gather information on farm size, bird type, bird age, history of antimicrobial use, and other poultry farm management practices in poultry farms. The questionnaire was pre-tested and administered through face-to-face interviews with one designated farm attendant from each of the 22 farms. For the current study, different samples were collected, including animal samples (chicken cloacal swabs), pooled environmental samples (boot swabs, poultry feed, and poultry drinking water), and pooled human samples (stool samples from farm handlers) (Figure 1).

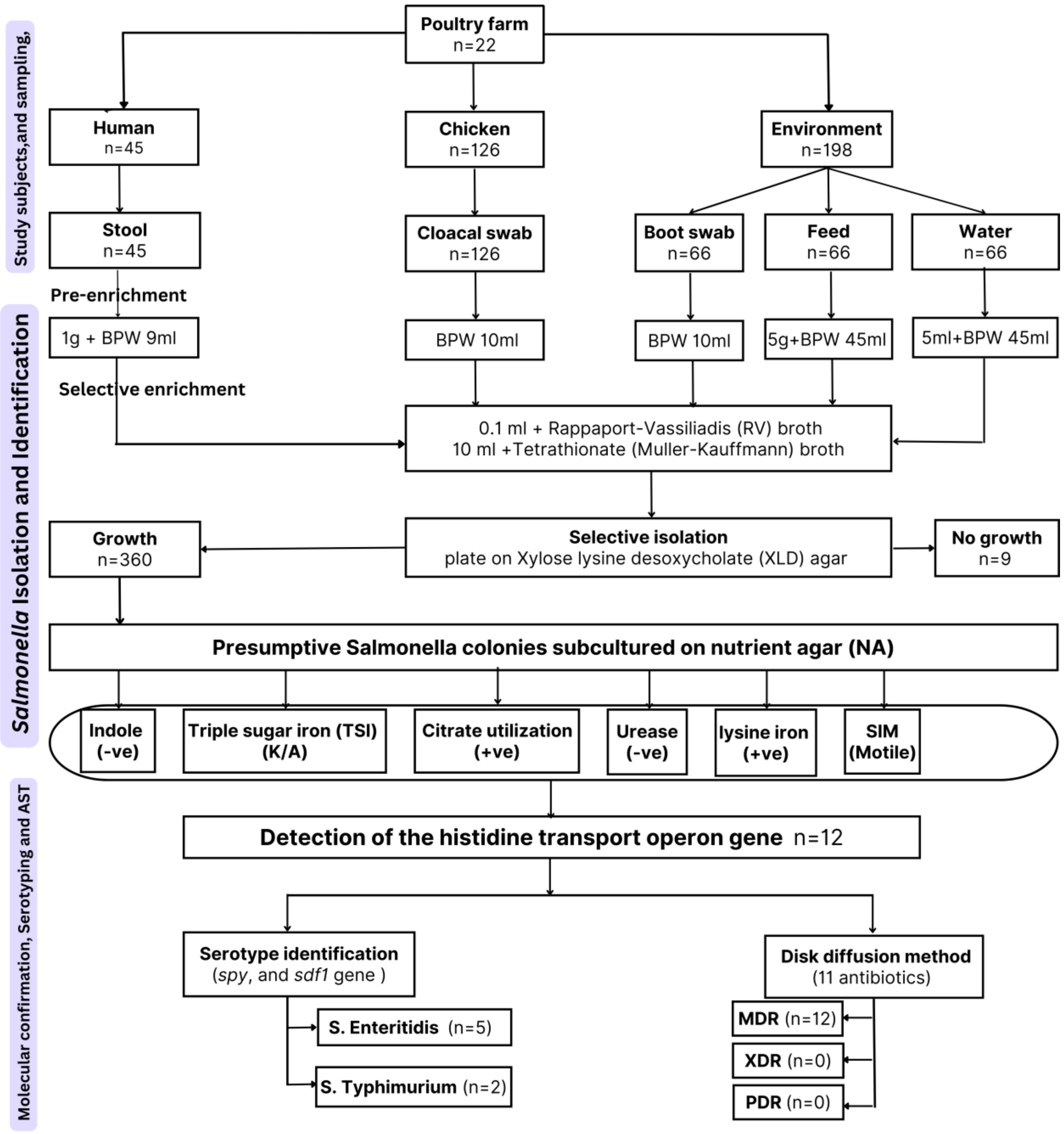

Figure 1. Schematic flowchart illustrating the study design, including sample collection, *Salmonella* isolation, biochemical and molecular confirmation, serotype identification, and antimicrobial susceptibility testing

Sample collection

A total of 369 samples were collected, comprising 126 cloacal swab specimens from chickens, 198 poultry-associated environmental samples, and 45 stool samples from volunteer farm attendants. The environmental samples included water (n = 66), feed (n = 66), and boot

swabs (n = 66), with three pooled samples collected per farm. Cloacal swab samples were collected using sterile cotton swabs containing 10 mL of buffered peptone water (BPW) (Oxoid, Basingstoke, UK) by gently rotating the swab within the cloaca of each bird. Boot swab samples were collected by wearing sterile plastic boots and walking on the poultry house floor. Poultry feed (5 g) and drinking water (5 mL) samples were collected from each farm using sterile zippered plastic bags and test tubes, respectively (30). Approximately 1 g of stool sample was collected from human volunteers using sterile stool cups with applicators (31). All samples were stored in iceboxes and transported under cold chain conditions to the microbiology laboratory unit of the Amhara Public Health Institute (APHI) for bacteriological analysis.

Isolation and molecular identification of *Salmonella* species and serotypes

The isolation and identification of non-typhoidal Salmonella (NTS) were conducted according to standard microbiological guidelines (32). Briefly, cloacal and boot swabs, feed (5 g), water (5 mL), and a stool (1 g) sample were pre-enriched in 10 mL, 45 mL, and 9 mL of BPW (Oxoid, Basingstoke, UK), respectively. All samples were vortex-mixed and incubated at 37 °C for 16–20 hours. Subsequently, 0.1 mL and 10 mL aliquots of the pre-enriched cultures were selectively enriched by inoculating them into 9.9 mL of Rappaport–Vassiliadis (RV) broth (Oxoid, Basingstoke, UK) at 41.5 °C for 24 hours and 10 mL of Muller–Kauffmann tetrathionate (MKTT) broth (HiMedia, Maharashtra, India) at 37 °C for 24 hours. Enriched cultures from both RV and MKTT broths were streaked onto xylose-lysine-desoxycholate (XLD) agar plates (HiMedia, Maharashtra, India) and incubated at 37 °C for 24–48 hours. Following incubation, the plates were examined for microbial growth, and colonies suspected to be *Salmonella* (pink with or without black centers) were selected. These colonies were subcultured onto nutrient agar (NA) plates (HiMedia, Maharashtra, India) and incubated at 37 °C for 18–24 hours. Isolates were further identified using a series of biochemical tests, including the indole test, triple sugar iron (TSI) test, citrate utilization test, urease test, lysine iron agar test, and sulfide-indole-motility (SIM) test as previously described (24) and interpreted according to the International Organization for Standardization guidelines.

Genus-level identification of presumptive *Salmonella* isolates was performed using uniplex PCR targeting the histidine transport operon gene, as previously described (33). Isolates confirmed as *Salmonella* spp. were further subjected to serotype identification by detecting the *S. Typhimurium*-specific gene (*spy*) (34) and *S. Enteritidis*-specific gene (*sdf1*) (35) (Figure 1).

In brief, bacterial DNA was extracted from overnight cultures grown on nutrient agar (NA) using a boiling lysis method. The quality and concentration of the extracted DNA were assessed using NanoDrop 2000/2000c spectrophotometers (Thermo Scientific, Boston, MA, USA). PCR assays were performed using a GeneAmp PCR System 2400 thermocycler (PerkinElmer, Shelton, CT, USA) in a 25-μL reaction volume. Each reaction contained 1 μL of DNA template, 12.5 μL of 2X PCR Master Mix (Promega, Madison, WI, USA) (containing 50 U/mL of Taq DNA polymerase, 3 mM MgCl2, and 400 μM each of dNTP mix), 0.5 μL of 0.2 μM of each primer (Bioneer, Daejeon, South Korea), and 10.5 μL of nuclease-free water.

The PCR cycling conditions included an initial denaturation at 94 °C for 5 minutes, followed by 30 cycles of denaturation (94 °C for 30 seconds), annealing (as shown in Table 1) for 30 seconds), and extension (72 °C for 30 seconds), with a final extension step (72 °C for 7 minutes). PCR products were analyzed by electrophoresis on 1.5% agarose gels (Bio Basic, Markham, ON, Canada) in 1× TAE buffer, stained with 1 µg/mL ethidium bromide. Gels were visualized and photographed using a gel documentation system (Bio-Rad, Hercules, CA, USA). A 100 bp DNA ladder (HiMedia, Maharashtra, India) was used as a molecular size marker. Primer sequences and annealing temperature used in the PCR assays are listed in Table 1.

Table 1. Oligonucleotide primer sequences and their annealing temperature used in PCR.

Primer use (Target Gene)	Nucleotide sequence (5′→3′)	Amplicon Size (bp)	Annealing (°C)	Reference
Salmonella enterica (*histidine transport operon*)	F: ACTGGCGTTATCCCTTTCTCTGGTG<br>R: ATGTTGTCCTGCCCCTGGTAAGAGA	496	54	(33)
S. Typhimurium (*spy*)	F: TTATTCACTTTTTACCCCTGAA<br>R: CCCTGACAG CCGTTAGATATT	401	52	(34)
S. Enteritidis (*sdf1*)	F: TGTGTTTTATCTGATGCAAGAGG<br>R: TGAACTACGTTCGTTCTTCTGG	304	55	(35)

Antimicrobial susceptibility testing

Antimicrobial susceptibility testing was performed using the Kirby–Bauer disk diffusion method on Muller–Hinton Agar (MHA) (HiMedia, Maharashtra, India) according to the Clinical and Laboratory Standards Institute (CLSI) guidelines (36). All *Salmonella* isolates were evaluated for susceptibility to 11 antibiotics representing eight antimicrobial classes: penicillins (ampicillin (10 μg)); cephalosporins (cephalothin (30 μg)); phenicols (chloramphenicol (30 μg)); fluoroquinolones (ciprofloxacin (5 μg), nalidixic acid (30 μg)); aminoglycosides (gentamycin (10 μg), streptomycin (10 μg), kanamycin (30 μg)); tetracyclines (tetracycline (30 μg)); sulfonamides (sulfisoxazole (1000 μg) and the folate pathway antagonist (sulfamethoxazole-trimethoprim (23.75/1.25 μg) (Oxoid, Basingstoke, UK). Antibiotic susceptibility was determined based on the breakpoints defined in CLSI M100 (36) and CLSI VET08 (37). Resistant *Salmonella* isolates were categorized as multidrug-resistant (MDR), extensively drug-resistant (XDR), and pan-drug-resistant (PDR) according to the criteria outlined in (38). The multiple antibiotic resistance index (MAR) was calculated using the following formula: MAR = a/b, where a represents the number of antibiotics to which the isolate was resistant, and b represents the total number of antibiotics tested in this study, as described in (39) (Figure 1).

Quality control and quality assurance

To ensure data quality and the reliability of the study findings, sample collection, transport, and storage strictly adhered to standard operating procedures. The sterility of the prepared media was verified by incubating plates for 24–48 h at 37 °C. To assess the accuracy of screening, confirmatory, and disk diffusion antimicrobial susceptibility tests, *S.* Enteritidis ATCC 13076 and *S.* Typhimurium ATCC 13311 (positive control) and *Escherichia coli* ATCC 25922 (negative control) were included alongside the tested isolates. These control strains were obtained from the Bacteriology, Parasitology, and Zoonosis Research Unit at the Ethiopian Public Health Institute (EPHI) in Addis Ababa, Ethiopia.

Data analysis

Data were initially entered and managed using Microsoft®Excel® 2019 (version 16.0.10414.20002; Microsoft Corporation, Redmond, WA, USA). The dataset was subsequently exported to STATA version 13.0 (StataCorp, College Station, TX, USA) for statistical analysis. Descriptive statistics were used to summarize the data. The prevalence of *Salmonella* on poultry farms was estimated as a percentage of farms with at least one positive sample-either from poultry or poultry-associated sources, relative to the total number of farms sampled. Associations between Salmonella occurrence and potential risk factors were assessed using the chi-square (χ^2) test. A p-value of <0.05 was considered indicative of statistical significance

Results

Farm characteristics

Poultry farms in this city are organized under Small and Micro-Enterprise offices. The majority (68.2%, 15/22) of farms focus primarily on egg production. All farms employed floor housing systems and implemented the all-in-all-out management principle, ensuring thorough cleaning and disinfection of poultry houses between flocks. Common clinical signs observed in these flocks included greenish-watery diarrhea, drooping heads, loss of appetite, bloody diarrhea, wing droop, and respiratory symptoms such as sneezing, coughing, and nasal discharge. Many farms also raised other domestic animals, including cattle and sheep, and lacked dedicated rodent control programs. Significant discrepancies were observed among the farms in feeding practices, water sources, and overall management strategies (Figure 2). While 32% of farms relied solely on commercially formulated feeds, 31.9% utilized a combination of commercial and on-farm-produced feeds. A concerning issue was the occurrence of sudden and unexpected flock mortality without any preceding signs of illness. Furthermore, a significant number of farm workers did not use adequate personal protective equipment (PPE).

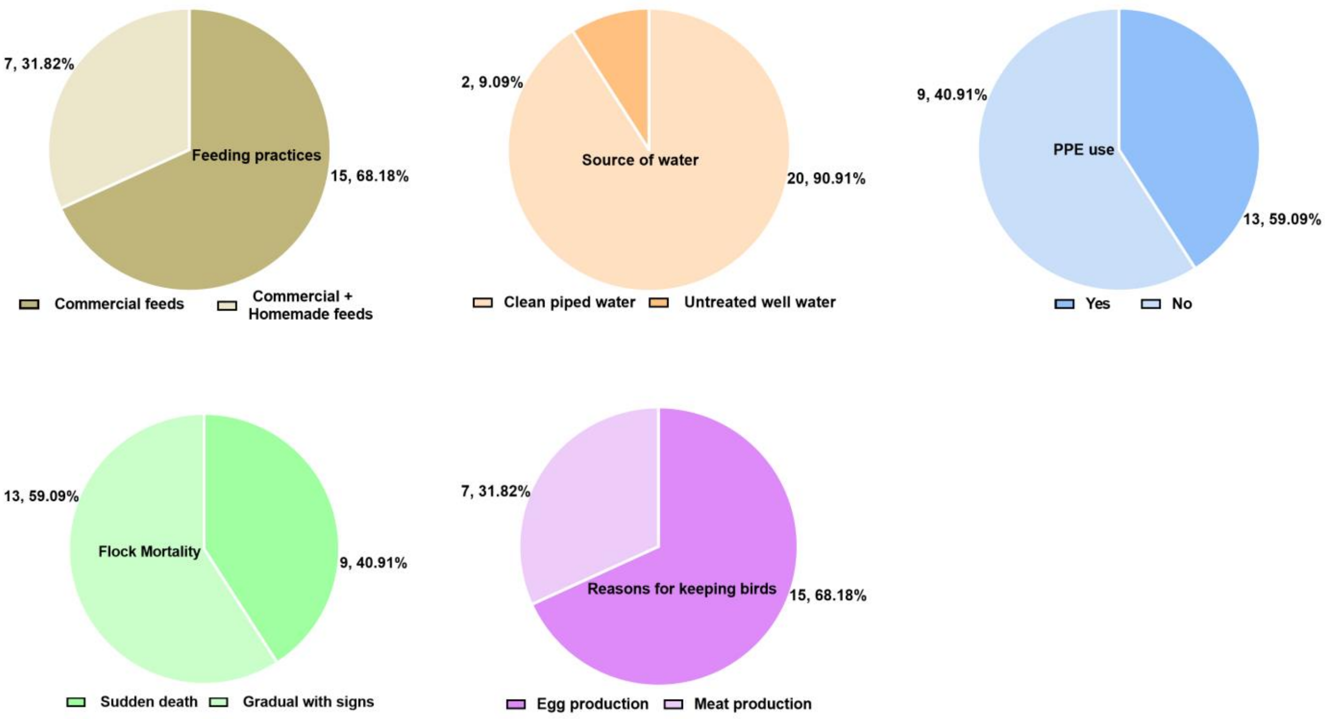

Figure 2: Feeding practices, water sources, biosecurity measures, and mortality rates on 22 poultry farms in northwestern Ethiopia

Prevalence of *Salmonella* isolates in poultry farms

NTS was identified in 18.2% (4/22) of poultry farms, including 20% (3/15) of layer farms and 14.3% (1/7) of broiler farms (Table 2). The prevalence of *Salmonella* (33.3%, 4/12) was significantly higher (p = 0.044) on large-scale farms compared to small-scale farms. Regarding the age of the flock, a higher proportion of positive samples for *Salmonella* spp. was observed in chickens aged 2–6 months (23.1%, 3/13). Neither production type nor flock age was significantly associated with the prevalence of *Salmonella* on poultry farms (Table 2). The study demonstrated a strong and statistically significant (p = 0.003) association between the use of disinfected boots and a lower prevalence of *Salmonella* on the farms. Interestingly, the presence of other animal species within the poultry farms was identified as a significant risk factor for *Salmonella* positivity (p = 0.001). The characteristics of the farms and the extent of *Salmonella* prevalence in relation to contributing risk factors are summarized in Table 2.

Table 2. Variation in *Salmonella* occurrence based on selected risk factors among poultry farms in Bahir Dar, Ethiopia.

Factors	No. of Farms	No. of Positive Farms (%)	Chi-Square	p-Value
Commodity type			0.105	0.746
Layers	15	3 (20.0)		
Broilers	7	1 (14.3)		
Flock size			4.074	0.044*
Small a	10	0 (0.0)		
Large b	12	4 (33.3)		
Age of chickens			1.109	0.775
<2 months	2	0 (0.0)		
2–6 months	13	3 (23.1)		
7–12 months	5	1 (20.0)		
>12 months	2	0 (0.0)		
Antimicrobial use			1.438	0.230
Yes	17	4 (23.5)		
No	5	0 (0.0)		
Foot bathing practice			8.556	0.003*
Yes	14	0 (0.0)		
No	8	4 (50.0)		
Presence of pests and insects			1.438	0.230
Yes	17	4 (23.5)		
No	5	0 (0.0)		
Presence of other livestock			9.900	0.002*
Yes	2	2 (100)		
No	20	2 (10)		
Supplementation of feed additives			0.014	0.905
Yes	17	3 (17.6)		
No	5	1 (20)		

Small a ≤1000; Large b >1000 birds, * = $p \leq 0.05$

Occurrence and serotypes of nontyphoidal *Salmonella* isolates based on sources

Of the samples tested, 3.3% (12/369) were positive for *Salmonella* species based on typical colonies, biochemical characteristics, and PCR detection of the histidine transport operon gene (S1 Figure). The prevalence of *Salmonella* in environmental boot samples (9.1%, 6/66) was significantly higher than in chicken cloacal samples (3.2%, 4/126) and human stool samples (4.4%, 2/45) (p-value of 0.031) (Table 3). PCR assays using serotype-specific genetic markers revealed the presence of two serotypes. Regardless of the isolate source, the most

prevalent serotype was *S.* Enteritidis at 41.6% (5/12), followed by *S.* Typhimurium at 16.6% (2/12). Untyped *Salmonella* strains accounted for 41.6% (5/12). *S. Enteritidis* was detected in all sample sources, with the highest prevalence in chicken samples. *S. Typhimurium* was only isolated from environmental samples (Table 3). All poultry feed and drinking water samples tested negative for *Salmonella* spp., *S.* Enteritidis, and *S.* Typhimurium. The overall and sample-wise prevalence of *Salmonella* is summarized in Table 3.

Table 3. Distribution of the most common *Salmonella* serotypes based on sample sources from poultry farms in Bahir Dar, Ethiopia.

Host	Samples	No. of Samples	*Salmonella* spp.	*S.* Enteritidis	*S.* Typhimurium	Untyped *Salmonella*
Chicken	Cloacal	126	4(3.2%)	2 (1.9%)	-	2(1.6%)
Enviroment	Boot	66	6 (9.1%)	2(3.0%)	2 (3.0%)	2(3.0%)
	Feed	66	-	-	-	-
	Water	66	-	-	-	-
Human	Stool	45	2(4.4%)	1 (2.2%)	-	1(2.2%)
	Total	369	12(3.3%)	5(1.4%)	2 (0.54%)	5(1.4%)

Antimicrobial susceptibility profile of *Salmonella* isolates

Figure 3 summarizes the results of the antimicrobial susceptibility testing of 11 antimicrobial agents belonging to eight different classes of antibiotics. *Salmonella* isolates from humans, chickens, and the environment exhibited 100% resistance to tetracycline and ampicillin, followed by 80% and 60% resistance to streptomycin and cephalothin, respectively. Moderate resistance to sulfisoxazole, nalidixic acid, and kanamycin was observed at a rate of 40%. In contrast, all isolates showed 100% sensitivity (low resistance) to gentamicin and ciprofloxacin (Figure 3a).

The resistance rate for the antibiotic agents against isolates from humans, chickens, and the environment was as follows: AMP (100%, 100%, and 100%), CEF (100%, 0%, and 100%), CHL (0%, 50%, and 0%), NAL (0%, 100%, and 0%), STR (100%, 50%, and 100%), KAN (0%, 50%, and 50%), TET (100%, 100%, and 100%), SFX (100%, 0%, and 50%), and SXT (0%, 50%) and 50%). Results revealed that *Salmonella* isolates from chickens exhibited unique resistance patterns, including resistance to nalidixic acid (n = 4) and chloramphenicol (n = 2).

In addition, resistance to ampicillin, streptomycin, and tetracycline was observed across all sample sources (Figure 3b).

All *Salmonella* isolates (100%) were classified as MDR, exhibiting resistance to three or more classes of antibiotics. *Salmonella* isolates from humans and the environment exhibited a common antimicrobial resistance pattern (33.3%), characterized by resistance to AMP, CEF, STR, TET, and SXT. The MAR index for all isolates exceeded 0.2, indicating a high level of resistance. The data showed that *S.* Typhimurium and *S.* Enteritidis isolates exhibited the highest MAR index value of 0.55, with six similar phenotypic antibiotic resistance patterns (AMP, CEF, STR, KAN, TET, and SFX). The most frequent MAR index observed was 0.45 (resistant to five out of eleven selected antibiotic agents) (Figure 3c). Extensively drug-resistant or pan-drug-resistant isolates were not recorded in the present study.

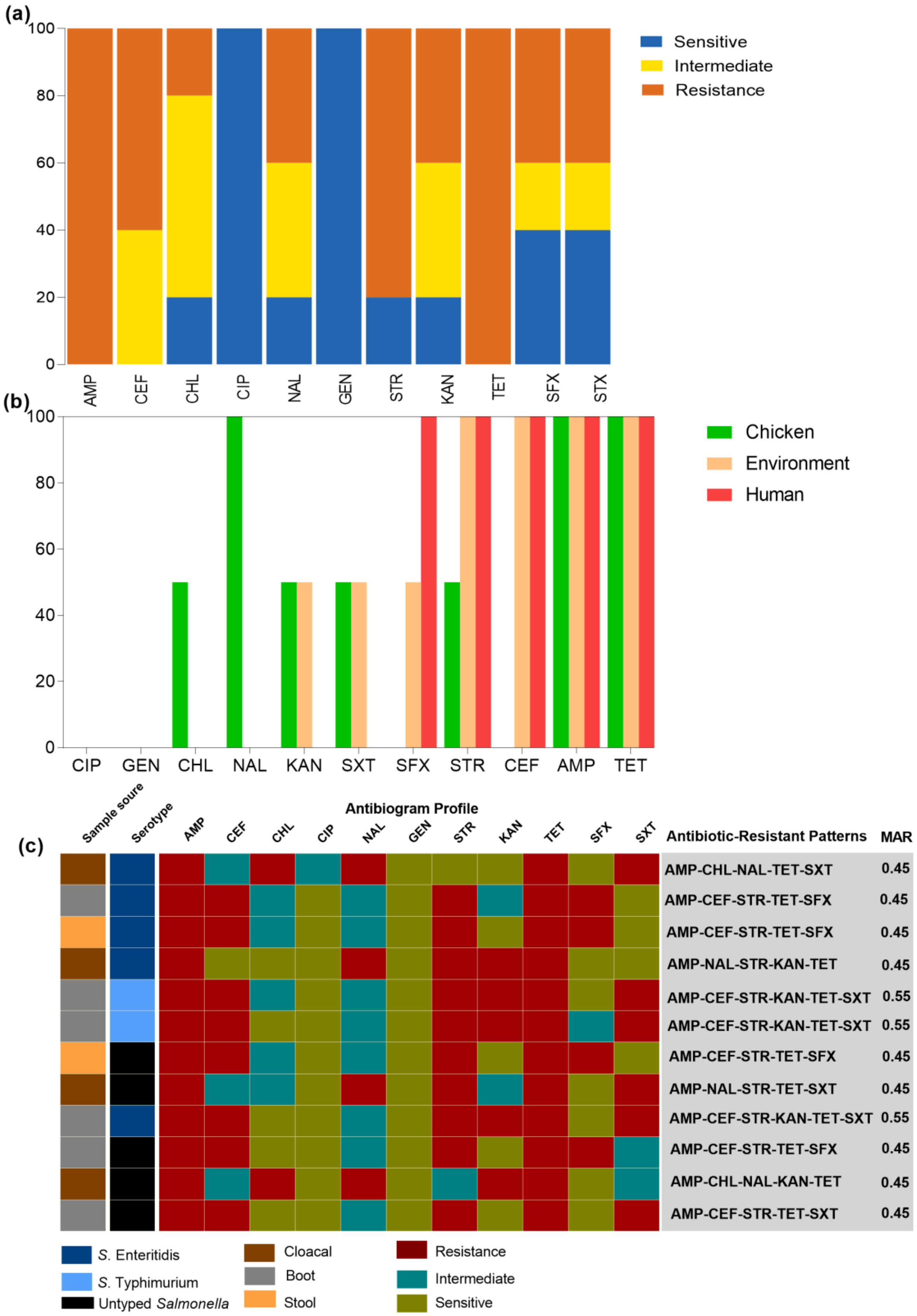
(a)
Sensitive
Intermediate
Resistance
100
80
60
40
20
0
AMP
CEF
CHL
CIP
NAL
GEN
STR
KAN
TET
SFX
STX
(b)
Chicken
Environment
Human
100
80
60
40
20
0
CIP
GEN
CHL
NAL
KAN
SXT
SFX
STR
CEF
AMP
TET
(c)
Sample soure
Serotype
Antibiogram Profile
AMP
CEF
CHL
CIP
NAL
GEN
STR
KAN
TET
SFX
SXT
Antibiotic-Resistant Patterns
MAR
AMP-CHL-NAL-TET-SXT 0.45
AMP-CEF-STR-TET-SFX 0.45
AMP-CEF-STR-TET-SFX 0.45
AMP-NAL-STR-KAN-TET 0.45
AMP-CEF-STR-KAN-TET-SXT 0.55
AMP-CEF-STR-KAN-TET-SXT 0.55
AMP-CEF-STR-TET-SFX 0.45
AMP-NAL-STR-TET-SXT 0.45
AMP-CEF-STR-KAN-TET-SXT 0.55
AMP-CEF-STR-TET-SFX 0.45
AMP-CHL-NAL-KAN-TET 0.45
AMP-CEF-STR-TET-SXT 0.45
S. Enteritidis
S. Typhimurium
Untyped Salmonella
Cloacal
Boot
Stool
Resistance
Intermediate
Sensitive

Figure 4. The phenotypic antimicrobial susceptibility profiles of *Salmonella* isolates from humans, chickens, and the environment in Bahir Dar, Ethiopia. (a) A column chart illustrates the antibiotic resistance rates observed among *Salmonella* isolates. b) The disparities in antibiotic resistance rates among *Salmonella* isolates from humans, chickens, and the environment. (c) Phenotypic antibiotic resistance patterns and multiple antibiotic resistance index of *Salmonella* isolates. AMP = ampicillin, CEF = cephalothin, CHL = chloramphenicol, CIP = ciprofloxacin, NAL = nalidixic acid, GEN = gentamycin, STR = streptomycin, KAN = kanamycin, TET = tetracycline, SFX = sulfisoxazole, SXT = trimethoprim/sulfamethoxazole.

Discussion

The poultry and livestock sectors are major contributors to the emergence and spread of antibiotic resistance, primarily due to the widespread and indiscriminate use of antibiotics for disease prevention and growth promotion. Poultry, in particular, serves as a significant reservoir of resistant bacteria and genes, posing a substantial risk to human health (40,41). In resource-limited countries, including Ethiopia, antimicrobial surveillance systems that evaluate the level of antibiotic resistance among priority pathogens are largely focused on healthcare facilities and human specimens (42). This narrow scope hinders a comprehensive understanding of antibiotic resistance and limits the effectiveness of control strategies.

In this study, we employed both conventional and molecular methods to investigate the prevalence and antibiotic resistance patterns of NTS isolates from poultry farms. To the best of our knowledge, this is the first study to document the incidence of NTS on poultry farms in northwestern Ethiopia. Our findings highlight that the farm environment, chickens, and farm workers in contact with poultry are potential sources of *Salmonella* infection. The study identified two circulating *Salmonella* serotypes on the investigated poultry farms, with the isolated NTS strains demonstrating multidrug resistance.

This cross-sectional study, conducted on commercial farms, revealed a direct effect of flock size, the use of boot dips, and the presence of other livestock on the occurrence of nontyphoidal Salmonellosis. The study provides a foundation for better understanding the transmission dynamics of infections and antibiotic resistance by examining three distinct

sample types. These findings are crucial for effectively addressing antimicrobial resistance (AMR) issues and guiding targeted prevention and control measures against *Salmonella* infection at national, regional, and global scales.

A recent study conducted in Bangladesh (43) and Uganda (44) reported a similar prevalence of NTS on poultry farms. In contrast, the current findings indicate a lower prevalence compared to comparable studies conducted in Nigeria (45), Algeria (46), and Nepal (47). *Salmonella* contamination was observed in 34.4%, 43.6%, and 55% of sampled farms, respectively. Another cross-sectional retrospective study in Brazil found a 32.1% prevalence of *Salmonella* across various commercial poultry sheds (48). However, the prevalence reported in this study is slightly higher than the previous findings, including a 14.6 % prevalence in poultry farms in central Ethiopia (24), 10. 6% in Indian livestock samples (including poultry cloacal swabs) (49), and 8 % in French broiler-chicken flocks (50). This study observed a higher prevalence of *Salmonella* spp. in the layer farms compared to the broiler farms. This finding aligns with another study that reported a higher prevalence of *Salmonella* in layers (46.2%) compared to broilers (41.3%) (51). The higher prevalence of *Salmonella* infection observed in laying hens may be attributed to the physiological stress associated with layers during egg production and molting. Such stress significantly impairs the immune response of layers, thereby increasing their susceptibility to *Salmonella* infection (52,53).

In this study, chickens aged 2–6 months showed a higher proportion of *Salmonella* spp. positive samples compared to older chickens. Chickens under two weeks of age are particularly susceptible to gastroenteritis and systemic disease caused by *Salmonella*. In contrast, adult hens often become asymptomatic carriers of *Salmonella enterica*. Although they show no overt symptoms, adult hens intermittently shed the bacteria in their feces (54). This study found no significant association between the prevalence of *Salmonella* on poultry farms and factors such as production type and age. These findings are consistent with previous studies conducted in Ethiopia, which similarly reported no statistically significant differences in *Salmonella* isolation rates across different production systems and age groups of birds (51,55).

This study demonstrates a significantly higher prevalence of *Salmonella* on large-scale poultry farms. The findings suggest that farms rearing larger flocks are at a higher risk of *Salmonella* contamination compared to those rearing smaller flocks. Indeed, evidence implies that flock size is a critical risk factor for the escalation of *Salmonella* contamination and infection (56,57). Poultry farms with high flock densities often encounter challenges related to overcrowding. Overcrowding can increase stress levels, reduce feed intake, and impair the immune response of birds. Massive flocks of chickens can also complicate the implementation of strict biosecurity measures and effective farm management practices. These factors collectively contribute to the increased occurrence and spread of *Salmonella* infection within flocks (58,59). Studies from Korea (60), Bangladesh (61), and Nigeria (62), also report a higher incidence of *Salmonella* spp. on poultry farms with large flocks.

The study identified a significant correlation between the use of disinfected boots, the presence of other livestock, and the prevalence of *Salmonella* on poultry farms. The external environment surrounding poultry houses emerged as a key pathway for the introduction and persistence of pathogens. These areas, often accessed by farm workers and visitors via their boots, can act as conduits for pathogen transmission. The implementation of boot dips containing disinfectant is crucial for mitigating the risk of *Salmonella* contamination and maintaining a safe poultry farming environment (63–65).

Furthermore, the findings suggest that other animals may serve as a source of nontyphoidal salmonellosis on poultry farms and their surrounding environments. *Salmonella* bacteria commonly reside in the gastrointestinal tracts of various animals, including livestock such as cattle, sheep, and pigs. These bacteria can be shed in the feces of both infected and healthy animals, making them potential reservoirs and sources of cross-contamination. Transmission can occur through direct contact with animals or indirectly via contaminated feed, water, equipment, or the surrounding environment (66,67). These results align with studies conducted in Uganda (44), Algeria (46), and Nigeria (62), which similarly reported a significant increase in the risk of *Salmonella* infection on farms where other animal species were present. Consequently, further research is necessary to comprehensively explore the role

of other animal species in the transmission of *Salmonella* on poultry farms. Such investigations could involve the identification of serotypes associated with these animals.

This study observed a low prevalence of *Salmonella* spp. in poultry-related samples compared to findings from other regions of Ethiopia. For instance, Abdi et al. (27) reported a 16.7% recovery rate of *Salmonella* from poultry and environmental samples in southern Ethiopia, while Asfaw Ali et al (68) reported a 14.6% prevalence in the cecal contents of exotic chickens in Debre Zeit. A study conducted on the East Coast of Peninsular Malaysia identified varying prevalence rates across different sources. Fecal samples exhibited the highest positivity rate at 59.5%, followed by cloacal swabs at 46.3%. Notably, sewage and tap water samples demonstrated significantly lower positivity rates (69). The *Salmonella* prevalence of 2.8% in Modjo and Adama (17), and 2.65% in Jimma, Ethiopia (70) are comparable to the findings of this study. In northern Poland, the prevalence of *Salmonella* spp in broiler chickens has gradually decreased from 2.19% in 2014 to 1.1% in 2015 and 1.22% in 2016 (71).

Interestingly, environmental boot samples demonstrated a higher prevalence of *Salmonella* than chicken cloacal swabs and human stool samples. This finding highlights the critical role of inadequate hygienic and poor biosecurity practices on poultry farms in the study area and suggests that boot or sock swabs are among the most effective methods for detecting *Salmonella* spp. in these environments (72). This aligns with a previous study conducted in Argentina, which also found that *Salmonella* spp. was most frequently detected on boot swabs, followed by fecal samples (73). Additionally, the presence of pests such as cockroaches, rodents, and insects may significantly contribute to the dissemination of *Salmonella* within poultry farms. These pests can serve as carriers of pathogens, leading to high levels of contamination in both poultry and the nearby environment (74). A concurrent study by Adesiyun et al. (58) revealed that 90% of farms with rat infestations exhibited the highest *Salmonella* contamination rates in their environment.

This study observed a lower recovery rate of *Salmonella* spp. from cloacal swabs, likely due to fluctuating bacterial concentrations in the cloacal openings, which are influenced by factors like age, stress, and health status. This inconsistency makes it challenging to isolate

Salmonella from cloacal samples. Moreover, cloacal samples may contain traces of feces or intestinal content, further complicating detection. Therefore, testing a large number of birds is essential to account for the intermittent shedding of *Salmonella* in feces and increase the likelihood of detecting its presence on farms (73,75).

Regarding stool samples from contact workers, this study reported a higher detection rate (6.7%) compared to a previous study in Adama, Ethiopia (2.8%) (17). In contrast, feed and drinking water samples tested negative for *Salmonella,* consistent with the findings of Djeffal et al. (46) and Abunna et al. (55). The observed variation in the *Salmonella* prevalence can be attributed to several factors, including geographical location, sample sizes, sample origin, sampling methods, and laboratory techniques. Additionally, the age of chickens, flock breed, farming systems, and biosecurity measures significantly influence the occurrence of *Salmonella* on poultry farms (76).

Poultry can be infected with various *Salmonella* serotypes, with *S*. Enteritidis and *S*. Typhimurium posing the highest public health concern. In recent years, the prevalence of *S*. Typhimurium has remained relatively stable across different farmed species, while *S*. Enteritidis has shown a significant increase in infections among both poultry and humans (77). Recent reports of infections and foodborne outbreaks are predominantly associated with contamination from multiple sources of poultry and poultry products contaminated with *S*. Enteritidis (78).

This study also identified *S*. Enteritidis and *S*. Typhimurium as the predominant serotypes. These serotypes have similarly been reported as the most prevalent serotypes in humans, poultry, and animal-derived food samples from Egypt (79), Pakistan (80), China (81), and Malaysia (82). However, contrary to these findings, a study from Nigeria indicated a limited role for *S*. Enteritidis and *S*. Typhimurium, with their absence observed on commercial poultry farms (45,62). Additionally, studies from other countries have reported the dominance of serotypes other than the commonly identified *S*. Enteritidis and *S*. Typhimurium in humans, poultry, and poultry products (44,83–85).

In this study, a substantial proportion of poultry farms (77.3%) reported using antimicrobials for various purposes, including feed additives, treatment, and disease prevention. This aligns with findings from a related study that revealed approximately 84% of livestock farmers in Ethiopia use antibiotics on their farms. Tetracycline, aminoglycosides, and sulfonamide-trimethoprim were identified as the most frequently employed antibiotic classes (86). The non-therapeutic use of antibiotics in animal agriculture, particularly in developing countries, is a growing concern. Antibiotics are often administered at sub-therapeutic doses as growth promoters to enhance livestock production and increase economic returns. However, this practice can inadvertently foster an environment that facilitates bacterial evolution and the emergence of antibiotic resistance (87).

The finding of relatively high resistance to tetracycline is consistent with studies conducted in India (88) and Turkey (89), which reported resistance rates of 100% and 93.34%, respectively. Sharma et al. (85) also demonstrated 100% resistance to tetracycline and 95.71% resistance to ampicillin in *Salmonella* isolates from retail chicken meat shops. Our results concur with previous findings that resistance to ampicillin, streptomycin, and tetracyclines was documented in *S.* Typhimurium strains isolated from diarrheic patients and food handlers in Kenya and northwestern Ethiopia (90,91). The high resistance rate to ampicillin and tetracycline is likely the prolonged and extensive use of these antimicrobials in veterinary medicine and the poultry industry. This continuous use exerts selective pressure, contributing significantly to the development and spread of antibiotic resistance. Conversely, all *Salmonella* isolates in this study demonstrated 100% sensitivity to gentamicin and ciprofloxacin, consistent with findings from Adama and Modjo, Ethiopia (17) and Erzurum, Turkey (89). However, these findings contrast with a previous report that indicated extensive drug resistance to ciprofloxacin in poultry and human samples from southern Ethiopia (27), Egypt (92), and Ghana (93). The absence of resistance to ciprofloxacin and gentamycin in this study suggests that these medications remain effective treatment options for *Salmonella* in poultry production within the study area. The relatively low resistance to ciprofloxacin may be attributed to its infrequent use, high cost, and limited availability. Furthermore, the low resistance to

gentamicin observed in *Salmonella* could be explained by the limited presence of inherent resistance mechanisms against aminoglycoside antibiotics.

In low-income countries, including Ethiopia, antimicrobials are often easily accessible without a prescription, resulting in indiscriminate use in poultry farming and human healthcare (94). This widespread use of antimicrobials in the poultry industry has likely exerted continuous selection pressure on *Salmonella*, contributing to the emergence of antimicrobial-resistant strains. In addition, *Salmonella* possesses intrinsic defense mechanisms that enable its survival and the development of antibiotic resistance. These mechanisms include the enzymatic inactivation of antibiotics, the expulsion of drugs from the cell via an efflux pump, the modification of drug targets, and the reduction of membrane permeability. Moreover, extensive dissemination of mobile antimicrobial resistance genes through horizontal gene transfer can occur within and beyond poultry farm environments. This allows bacteria to persist even in the absence of direct selective pressure (95,96). In this study, all *S.* Typhimurium and *S.* Enteritidis isolates exhibited MDR patterns with MAR index ranging from 0.45 to 0.55 across different sample sources. MAR index values exceeding 0.2 typically indicate that the isolates originate from high-risk, heavily contaminated environments where antibiotic use is frequent (97). The occurrence of MDR *Salmonella* underscores the potential role of chickens and poultry farms as significant reservoirs for human pathogens and antibiotic-resistant genes.

This study was subject to limitations, including its cross-sectional nature, exclusion of additional sources such as air, poultry products, and by-products, and inability to detect virulence and antibiotic resistance genes. Despite these limitations, this study underscores the role of the poultry industry in antimicrobial resistance dynamics and disease epidemiology within the broader regional context of the One Health framework. Longitudinal studies that incorporate appropriate sample size calculations, with a particular focus on diverse sample sources and farm-level clustering of chickens, could enhance our understanding of patterns of *Salmonella* transmission and evolution within and between farms. Additionally, such studies would allow a more comprehensive identification of farm-specific risk factors for *Salmonella* prevalence and antimicrobial resistance across sample sources. Future research should

integrate fundamental microbiology with high-throughput multi-omics approaches to uncover the key drivers of antimicrobial resistance, elucidate the mechanisms of horizontal gene transfer, evaluate environmental influences and reservoirs, and map evolutionary pathways. Understanding these determinants can facilitate the development of novel strategies that extend beyond traditional antimicrobial stewardship efforts. Moreover, continuous evaluations of phenotypic resistance and the ability of the *Salmonella* strains to horizontally transfer resistance genes through minimum inhibitory concentration (MIC) determination and conjugation assays are substantial for accurately characterizing the bona fide resistance patterns in these strains.

Conclusions

In summary, this study underscores the alarming prevalence of multidrug-resistant nontyphoidal *Salmonella* on poultry farms in northwestern Ethiopia, posing a significant public health threat. All *Salmonella* isolates exhibited complete resistance to tetracyclines and penicillins, antibiotics frequently used in veterinary practice. To effectively combat antibiotic resistance in this region, further efforts should focus on establishing an integrated surveillance system, promoting the prudent use of critical antibiotics, and exploring the potential of drug combinations and natural antimicrobial agents. *S.* Enteritidis emerged as the predominant serotype in chickens, humans, and farm environments. Given the zoonotic nature of these serotypes, high-resolution microbial genomics is essential for understanding their transmission dynamics and identifying sources of *Salmonella* serotypes between humans and animals. Moreover, large flock sizes, the presence of other animal species, and improper foot baths were identified as potential risk factors for the spread of nontyphoidal salmonellosis. Therefore, implementing robust biosecurity measures, effective flock management practices, and controlling the presence of other animal species are essential for mitigating the spread of nontyphoidal salmonellosis in this region.

Supporting information

S1 Figure. Raw images during the study period, including sample collection, Salmonella isolation, confirmation, serotyping, and antimicrobial susceptibility testing

Acknowledgments

The authors would like to express their sincere gratitude to the farm owners and the Bahir Dar Agricultural Development Office for their cooperation and for providing the essential information for this study. We also extend our appreciation to the Microbiology Laboratory Unit of the Amhara Public Health Institute for their valuable technical assistance during laboratory activities throughout the experiment.

Author contributions

ABM: Conceptualization, Data curation, Formal analysis, Investigation, Methodology, Writing original draft, Writing review and editing. MAB: Conceptualization, Investigation Methodology, Validation, Supervision, Writing original draft, Writing review and editing. HT: Conceptualization, Methodology, Supervision, Validation, Writing original draft, Writing review and editing. H.Y.K: Conceptualization, Data curation, Formal analysis, Methodology, Writing review and editing. B.G.A: Conceptualization, Methodology, Supervision, Validation, Writing review, and editing. HCT: Conceptualization, Data curation, Formal analysis, Methodology, Writing review and editing. DA: Conceptualization, Methodology, Supervision, Validation, Writing review and editing. All authors have read and agreed to the published version of the manuscript.

References

1. Galanis E, Lo Fo Wong DMA, Patrick ME, Binsztein N, Cieslik A, Chalermchaikit T, et al. Web-based Surveillance and Global Salmonella Distribution, 2000–2002. Emerg Infect Dis. 2006;12(3):381–8. https://doi.org/10.3201/eid1205.050854.
2. Eng SK, Pusparajah P, Ab Mutalib NS, Ser HL, Chan KG, Lee LH. Salmonella: A review on pathogenesis, epidemiology, and antibiotic resistance. Front Life Sci. 2015;8(3):284–93. https://doi.org/10.1080/21553769.2015.1051243.
3. Majowicz SE, Musto J, Scallan E, Angulo FJ, Kirk M, O’Brien SJ, et al. The global burden of nontyphoidal Salmonella gastroenteritis. Clin Infect Dis. 2010;50(6):882–9. https://doi.org/10.1086/650733.
4. Gilchrist JJ, MacLennan CA. Invasive Nontyphoidal Salmonella Disease in Africa. Am. Soc. Microbiol. 2019(8):10-1128. https://doi.org/10.1128/ecosalplus.esp-0007-2018.
5. Tadesse G. Prevalence of human Salmonellosis in Ethiopia: a systematic review and meta-analysis. BMC Infect Dis. 2014;14:88.https://doi.org/10.1186/1471-2334-14-88.
6. Hugas MhM, Beloeil PA. Controlling Salmonella along the food chain in the European Union-progress over the last ten years. Euro surveillance. 2014;19(19):20804. https://doi.org/10.2807/1560-7917.ES2014.19.19.20804.
7. Zhang S, Li S, Gu W, Den Bakker H, Boxrud D, Taylor A, et al. Zoonotic source attribution of Salmonella enterica serotype Typhimurium using genomic surveillance data, United States. Emerg Infect Dis. 2019;25(1):82–91. https://doi.org/10.3201/eid2501.180835.
8. Li S, He Y, Mann DA, Deng X. Global spread of Salmonella Enteritidis via centralized sourcing and international trade of poultry breeding stocks. Nat Commun. 2021;12(1). 5109. https://doi.org/10.1038/s41467-021-25319-7
9. Andino A, Hanning I. Salmonella enterica: Survival, colonization, and virulence differences among serovars. Scientific World Journal. 2015;2015:520179. https://doi.org/10.1155/2015/520179.
10. Carrasco E, Morales-Rueda A, García-Gimeno RM. Cross-contamination and recontamination by Salmonella in foods: A review. Food Research International. 2012;45(2):545–56. https://doi.org/10.1016/j.foodres.2011.11.004
11. Centers for Disease Control and Prevention (CDC). Surveillance for Foodborne Disease Outbreaks, United States, 2017, Annual Report. Atlanta, Georgia: U.S. Department of Health and Human Services, CDC, 2019. Available online: https://www.cdc.gov/fdoss/pdf/2017_FoodBorneOutbreaks_508.pdf (accessed on 21 June 2021).
12. Wang W, Cui J, Liu F, Hu Y, Li F, Zhou Z, et al. Genomic characterization of Salmonella isolated from retail chicken and humans with diarrhea in Qingdao, China. Front Microbiol. 2023;18(14):1295769. https://doi.org/10.3389/fmicb.2023.1295769.

13. Hoelzer K, Switt AIM, Wiedmann M. Animal contact as a source of human non-typhoidal salmonellosis. Vet Res. 2011;42(1):34. https://doi.org/10.1186/1297-9716-42-34.
14. Pornsukarom S, Van Vliet AHM, Thakur S. Whole genome sequencing analysis of multiple Salmonella serovars provides insights into phylogenetic relatedness, antimicrobial resistance, and virulence markers across humans, food animals, and agriculture environmental sources. BMC Genomics. 2018;19(1):801. https://doi.org/10.1186/s12864-018-5137-4.
15. Akinyemi KO, Fakorede CO, Linde J, Methner U, Wareth G, Tomaso H, et al. Whole genome sequencing of Salmonella enterica serovars isolated from humans, animals, and the environment in Lagos, Nigeria. BMC Microbiol. 2023;23(1):164. https://doi.org/10.1186/s12866-023-02901-1.
16. Cheong HJ, Lee YJ, Hwang IS, Kee SY, Cheong HW, Song JY, et al. Characteristics of Non-typhoidal Salmonella Isolates from Human and Broiler Chickens in Southwestern Seoul, Korea. J Korean Med Sci. 2007;22(5):773–8. https://doi.org/10.3346/jkms.2007.22.5.773.
17. Dagnew B, Alemayehu H, Medhin G, Eguale T. Prevalence and antimicrobial susceptibility of Salmonella in poultry farms and in-contact humans in Adama and Modjo towns, Ethiopia. Microbiology open. 2020;9(8): e1067. https://doi.org/10.1002/mbo3.1067.
18. Marshall BM, Levy SB. Food animals and antimicrobials: Impacts on human health. Clin Microbiol Rev. 2011;24(4):718–33. https://doi.org/10.1128/CMR.00002-11.
19. Karimiazar F, Soltanpour MS, Aminzare M, Hassanzadazar H. Prevalence, genotyping, serotyping, and antibiotic resistance of isolated Salmonella strains from industrial and local eggs in Iran. J Food Saf. 2019;39(1):e12585. https://doi.org/10.1111/jfs.12585.
20. Van TTH, Yidana Z, Smooker PM, Coloe PJ. Antibiotic use in food animals worldwide, with a focus on Africa: Pluses and minuses. J Glob Antimicrob Resist. 2020;20:170–7. https://doi.org/10.1016/j.jgar.2019.07.031.
21. Kumar D, Pornsukarom S, Thakur S. Antibiotic Usage in Poultry Production and Antimicrobial-Resistant Salmonella in Poultry. In: Food Safety in Poultry Meat Production. Springer International Publishing; Berlin/Heidelberg, Germany, 2019; pp. 47–66. https://doi.org/10.1007/978-3-030-05011-5_3.
22. Delgado-Suárez EJ, Palós-Guitérrez T, Ruíz-López FA, Hernández Pérez CF, Ballesteros-Nova NE, Soberanis-Ramos O, et al. Genomic surveillance of antimicrobial resistance shows cattle and poultry are a moderate source of multi-drug resistant nontyphoidal Salmonella in Mexico. PLoS One. 2021;16(5):e0243681. https://doi.org/10.1371/journal.pone.0243681.
23. Dieye Y, Hull DM, Wane AA, Harden L, Fall C, Sambe-Ba B, et al. Genomics of human and chicken Salmonella isolates in Senegal: Broilers as a source of antimicrobial resistance and potentially invasive nontyphoidal salmonellosis infections. PLoS One. 2022;17(3):e0266025. https://doi.org/10.1371/journal.pone.0266025.

24. Eguale T. Non-typhoidal Salmonella serovars in poultry farms in central Ethiopia: Prevalence and antimicrobial resistance. BMC Vet Res. 2018;14:1–8. https://doi.org/10.1186/s12917-018-1539-4.
25. Asefa Kebede I, Duga T. Prevalence and Antimicrobial Resistance of Salmonella in Poultry Products in Central Ethiopia. Vet Med Int. 2022;2022:8625636. https://doi.org/10.1155/2022/8625636.
26. Mohammed Y, Dubie T. Isolation, identification and antimicrobial susceptibility profile of Salmonella isolated from poultry farms in Addis Ababa, Ethiopia. Vet Med Sci. 2022;8(3):1166–73. https://doi.org/10.1002/vms3.762.
27. Abdi RD, Mengstie F, Beyi AF, Beyene T, Waktole H, Mammo B, et al. Determination of the sources and antimicrobial resistance patterns of Salmonella isolated from the poultry industry in Southern Ethiopia. BMC Infect Dis. 2017;17:1–12. https://doi.org/10.1186/s12879-017-2437-2.
28. Mogasale V, Maskery B, Ochiai L, Lee JS, Mogasale V V, Ramani E, et al. Burden of typhoid fever in low-income and middle-income countries: a systematic, literature-based update with risk-factor adjustment. Lancet Glob Health. 2014;2(10):e570–e580. https://doi.org/10.1016/s2214-109x(14)70301-8.
29. Abebe G. Long-term climate data description in Ethiopia. Data Brief. 2017;14:371–92. https://doi.org/10.1016/j.dib.2017.07.052.
30. Alali WQ, Thakur S, Berghaus RD, Martin MP, Gebreyes WA. Prevalence and distribution of Salmonella in organic and conventional broiler poultry farms. Foodborne Pathog Dis. 2010;7(11):1363–71. https://doi.org/10.1089/fpd.2010.0566.
31. Lamboro T, Ketema T, Bacha K. Prevalence and Antimicrobial Resistance in Salmonella and Shigella Species Isolated from Outpatients, Jimma University Specialized Hospital, Southwest Ethiopia. Can. J. Infect Dis Med Microbiol. 2016;2016(1):4210760. https://doi.org/10.1155/2016/4210760.
32. ISO 6579-1. Microbiology of the food chain — Horizontal method for the detection, enumeration and serotyping of Salmonella Part 1: Detection of Salmonella spp. Edition 1. ISO. Geneva, Switzerland; 2017. Available online: https://www.iso.org/obp/ui/#iso:std:iso:6579:-1:ed-1:v1:en (accessed on 23 June 2021). https://doi.org/10.1177/104063879300500311.
33. Cohen ND, Neibergs HL, Mcgruder ED, Whitford HW, Behle RW, Ray PM, et al. Genus-specific detection of salmonellae using the polymerase chain reaction (PCR). J Vet Diagn Invest. 1993;5(3):368–71.
34. Alvarez J, Sota M, Vivanco AB, Perales I, Cisterna R, Rementeria A, et al. Development of a Multiplex PCR Technique for Detection and Epidemiological Typing of *Salmonella* in Human Clinical Samples. J Clin Microbiol. 2004;42(4):1734–8. https://doi.org/10.1128/jcm.42.4.1734-1738.2004.

35. de Freitas CG, Santana ÂP, da Silva PHC, Gonçalves VSP, Barros M de AF, Torres FAG, et al. PCR multiplex for detection of Salmonella Enteritidis, Typhi and Typhimurium and occurrence in poultry meat. Int J Food Microbiol. 2010;139(1–2):15–22. https://doi.org/10.1016/j.ijfoodmicro.2010.02.007.
36. Clinical and Laboratory Standards Institute (CLSI). Performance Standards for Antimicrobial Susceptibility Testing. 30th ed. Supplement M100. Clinical and Laboratory Standards Institute. Wayne, PA, USA; 2020.
37. Clinical Laboratory Standards Institute (CLSI). Performance standards for antimicrobial disk and dilution susceptibility tests for bacteria isolated from animals. 4th ed. CLSI Supplement VET08. Clinical Laboratory Standards Institute. Wayne, PA, USA; 2018.
38. Magiorakos AP, Srinivasan A, Carey RB, Carmeli Y, Falagas ME, Giske CG, et al. Multidrug-resistant, extensively drug-resistant and pandrug-resistant bacteria: An international expert proposal for interim standard definitions for acquired resistance. Clinical Microbiology and Infection. 2012;18(3):268–81. https://doi.org/10.1111/j.1469-0691.2011.03570.x.
39. Krumperman PH. Multiple Antibiotic Resistance Indexing of Escherichia coli to Identify High-Risk Sources of Fecal Contamination of Foods. Appl Environ Microbiol. 1983;46(1):165–70. https://doi.org/10.1128/aem.46.1.165-170.1983.
40. Chowdhury S, Ghosh S, Aleem MA, Parveen S, Islam MA, Rashid MM, et al. Antibiotic usage and resistance in food animal production: What have we learned from Bangladesh? Antibiotics. 2021;10(1032):1–14.https://doi.org/10.3390/antibiotics10091032.
41. Nhung NT, Chansiripornchai N, Carrique-Mas JJ. Antimicrobial resistance in bacterial poultry pathogens: A review. Front Vet Sci. 2017;4(126):126. https://doi.org/10.3389/fvets.2017.00126.
42. Ibrahim RA, Teshale AM, Dinku SF, Abera NA, Negeri AA, Desta FG, et al. Antimicrobial resistance surveillance in Ethiopia: Implementation experiences and lessons learned. Afr J Lab Med. 2018;7(2):1–4. https://doi.org/10.4102/ajlm.v7i2.770.
43. Barua H, Biswas PK, Olsen KEP, Christensen JP. Prevalence and characterization of motile Salmonella in commercial layer poultry farms in Bangladesh. PLoS One. 2012;7(4):e35914. https://doi.org/10.1371/journal.pone.0035914.
44. Odoch T, Wasteson Y, L'Abée-Lund T, Muwonge A, Kankya C, Nyakarahuka L, et al. Prevalence, antimicrobial susceptibility and risk factors associated with non-typhoidal Salmonella on Ugandan layer hen farms. BMC Vet Res. 2017;13(1):365. https://doi.org/10.1186/s12917-017-1291-1.
45. Fagbamila IO, Barco L, Mancin M, Kwaga J, Ngulukun SS, Zavagnin P, et al. Salmonella serovars and their distribution in Nigerian commercial chicken layer farms. PLoS One. 2017;12(3):e0173097. https://doi.org/10.1371/journal.pone.0173097.

46. Djeffal S, Mamache B, Elgroud R, Hireche S, Bouaziz O. Prevalence and risk factors for Salmonella spp. contamination in broiler chicken farms and slaughterhouses in the northeast of Algeria. Vet World. 2018;11(8):1102–8. https://doi.org/10.14202/vetworld.2018.1102-1108.
47. Sharma S, Fowler PD, Pant DK, Singh S, Wilkins MJ. Prevalence of non-typhoidal Salmonella and risk factors on poultry farms in Chitwan, Nepal. Vet World. 202;14(2):426–36. https://doi.org/10.14202/vetworld.2021.426-436.
48. Silva ND da, Laurindo EE, Martins CM, Silveira RMP, Silveira CT da, Santin E. Association between non-typhoidal Salmonella isolated from commercial poultry sheds and associated factors in Paraná, Brazil: Cross-sectional retrospective study. Arq Inst Biol. 2021;88(e00402020):1–20. https://doi.org/10.1590/1808-1657000402020.
49. Sudhanthirakodi S, Jain P, Chattopadhyay UK, Dutta S. Non-typhoidal Salmonella isolates from livestock and food samples, Kolkata, India. Journal of Microbiology and Infectious Diseases. 2016;6(3):113–20. https://doi.org/10.5799/jmid.vi.328974.
50. Le Bouquin S, Allain V, Rouxel S, Petetin I, Picherot M, Michel V, et al. Prevalence and risk factors for Salmonella spp. contamination in French broiler-chicken flocks at the end of the rearing period. Prev Vet Med. 2010;97(3):245–51. https://doi.org/10.1016/j.prevetmed.2010.09.014.
51. Alebachew K, Mekonnen A. A survey on Salmonella infection among chicken flocks in Jimma town, Ethiopia. Afr J Microbiol Res. 2013;7(14):1239–45. https://doi.org/10.14202/vetworld.2018.1102-1108.
52. Landers KL, Woodward CL, Li X, Kubena LF, Nisbet DJ, Ricke SC. Alfalfa as a single dietary source for molt induction in laying hens. Bioresour Technol. 2005;96(5):565–70. https://doi.org/10.1016/j.biortech.2004.06.013.
53. Gole VC, Caraguel CGB, Sexton M, Fowler C, Chousalkar KK. Shedding of Salmonella in single age caged commercial layer flock at an early stage of lay. Int J Food Microbiol. 2014;189:61–6. https://doi.org/10.1016/j.ijfoodmicro.2014.07.030.
54. Shah DH, Casavant C, Hawley Q, Addwebi T, Call DR, Guard J. Salmonella enteritidis strains from poultry exhibit differential responses to acid stress, oxidative stress, and survival in the egg albumen. Foodborne Pathog Dis. 2012;9(3):258–64. https://doi.org/10.1089/fpd.2011.1009.
55. Abunna F, Ashenafi D, Beyene T, Ayana D, Mamo B, Duguma R. Isolation, identification and antimicrobial susceptibility profiles of *Salmonella* isolates from dairy farms in and around Modjo town, Ethiopia. Ethiop Vet J. 2017;21(2):92–108. https://doi.org/10.4314/evj.v21i2.7.
56. Chemaly M, Huneau-Salau¨n A, Salau¨n S, Labbe A, Houdayer C, Petetin I, et al. Isolation of Salmonella enterica in Laying-Hen Flocks and Assessment of Eggshell Contamination in France. J Food Prot. 2009;72(10):2071–7. https://doi.org/10.4315/0362-028x- 72.10.2071.

57. Snow LC, Davies RH, Christiansen KH, Carrique-Mas JJ, Cook AJC, Evans SJ. Investigation of risk factors for Salmonella on commercial egg-laying farms in Great Britain, 2004-2005. Veterinary Record. 2010;166(19):579–86. https://doi.org/10.1136/vr.b4801.
58. Adesiyun A, Webb L, Musai L, Louison B, Joseph G, Stewart-Johnson A, et al. Survey of salmonella contamination in chicken layer farms in three Caribbean countries. J Food Prot. 2014;77(9):1471–80. https://doi.org/10.4315/0362- 028x.jfp-14-021.
59. Abo-Al-Ela HG, El-Kassas S, El-Naggar K, Abdo SE, Jahejo AR, Al Wakeel RA. Stress and immunity in poultry: light management and nanotechnology as effective immune enhancers to fight stress. Cell Stress Chaperones. Cell Stress Chaperones. 2021;26:457–72. https://doi.org/10.1007/s12192-021-01204-6.
60. Im MC, Jeong SJ, Kwon YK, Jeong OM, Kang MS, Lee YJ. Prevalence and characteristics of Salmonella spp. isolated from commercial layer farms in Korea. Poult Sci. 2015;94(7):1691–8. https://doi.org/10.3382/ps/pev137.
61. Sarker BR, Ghosh S, Chowdhury S, Dutta A, Chandra Deb L, Krishna Sarker B, et al. Prevalence and antimicrobial susceptibility profiles of non-typhoidal Salmonella isolated from chickens in Rajshahi, Bangladesh. Vet Med Sci. 2021;7(3):820–30. https://doi.org/10.1002/vms3.440.
62. Jibril AH, Okeke IN, Dalsgaard A, Kudirkiene E, Akinlabi OC, Bello MB, et al. Prevalence and risk factors of Salmonella in commercial poultry farms in Nigeria. PLoS One. 2020;15(9):e0238190. https://doi.org/10.1371/journal.pone.0238190.
63. Wang J, Vaddu S, Bhumanapalli S, Mishra A, Applegate T, Singh M, et al. A systematic review and meta-analysis of the sources of Salmonella in poultry production (pre-harvest) and their relative contributions to the microbial risk of poultry meat. Poult Sci. 2023;102(5):102566. https://doi.org/10.1016/j.psj.2023.102566.
64. Rashid T, VonVille HM, Hasan I, Garey KW. Shoe soles as a potential vector for pathogen transmission: a systematic review. J Appl Microbiol. 2016;121(5):1223–31. https://doi.org/10.1111/jam.13250.
65. Rabie AJ, McLaren IM, Breslin MF, Sayers R, Davies RH. Assessment of anti-Salmonella activity of boot dip samples. 2015;44(2):129–34.https://doi.org/10.1080/03079457.2015.1012046.
66. Munck N, Smith J, Bates J, Glass K, Hald T, Kirk MD. Source Attribution of Salmonella in Macadamia Nuts to Animal and Environmental Reservoirs in Queensland, Australia. Foodborne Pathog Dis. 2020;17(5):357–64. https://doi.org/10.1089/fpd.2019.2706.
67. Parisi A, Phuong TLT, Mather AE, Jombart T, Tuyen HT, Lan NPH, et al. The role of animals as a source of antimicrobial resistant nontyphoidal Salmonella causing invasive and non-invasive human disease in Vietnam. Infection, Genetics and Evolution. 2020;85:104534. https://doi.org/10.1016/j.meegid.2020.104534.

68. Asfaw Ali D, Tadesse B, Ebabu A. Prevalence and Antibiotic Resistance Pattern of Salmonella Isolated from Caecal Contents of Exotic Chicken in Debre Zeit and Modjo, Ethiopia. Int J Microbiol. 2020;2020:1–6. https://doi.org/10.1155/2020/1910630.
69. Osman AY, Elmi SA, Simons D, Elton L, Haider N, Azam Khan M, et al. Antimicrobial resistance patterns and risk factors associated with salmonella spp. Isolates from poultry farms in the east coast of peninsular Malaysia: A cross-sectional study. Pathogens. 2021 Sep 1;10(9):1160. https://doi.org/10.3390/pathogens10091160.
70. Taddese D, Tolosa T, Deresa B, Lakow M, Olani A, Shumi E. Antibiograms and risk factors of Salmonella isolates from laying hens and eggs in Jimma Town, South Western Ethiopia. BMC Res Notes. 2019;12(472):1–7. https://doi.org/10.1186/s13104-019- 4516-5.
71. Witkowska D, Kuncewicz M, Żebrowska JP, Sobczak J, Sowińska J. Prevalence of salmonella spp. In broiler chicken flocks in northern Poland in 2014–2016. Annals of Agricultural and Environmental Medicine. 2018;25(4):693–7. https://doi.org/10.26444/aaem/99528.
72. Crabb HK, Allen JL, Devlin JM, Wilks CR, Gilkerson JR. Spatial Distribution of Salmonella enterica in Poultry Shed Environments Observed by Intensive Longitudinal Environmental Sampling. Appl Environ Microbiol. 2019;85:e00333-19. https://doi.org/10.1128/aem.00333-19.
73. Soria MC, Soria MA, Bueno DJ, Godano EI, Gómez SC, ViaButron IA, et al. Salmonella spp. contamination in commercial layer hen farms using different types of samples and detection methods. Poult Sci. 2017;96(8):2820–30. https://doi.org/10.3382/ps/pex053.
74. Yang H, Dey SK, Buchanan R, Biswas D. Pests in Poultry, Poultry Product Borne Infection and Future Precautions. In Practical Food Safety: Contemporary Issues and Future Directions. First Edition. New Jersey, United States: John Wiley & Sons, Ltd.; 2014. 535–552.
75. Mueller-Doblies D, Sayers AR, Carrique-Mas JJ, Davies RH. Comparison of sampling methods to detect Salmonella infection of turkey flocks. J Appl Microbiol. 2009;107(2):635–45. https://doi.org/10.1111/j.1365-2672.2009.04230.x.
76. Ramtahal MA, Amoako DG, Akebe ALK, Somboro AM, Bester LA, Essack SY. A Public Health Insight into Salmonella in Poultry in Africa: A Review of the Past Decade: 2010-2020. Microbial Drug Resistance. 2022;28(6):710–33. https://doi.org/10.1089/mdr.2021.0384.
77. Salmonellosis, S. Enteritidis and S. typhimurium Infections. 2019. Available online: https://www.thepoultrysite.com/diseaseguide/salmonellosis-s-enteritidis-and-s-typhimurium-infections (accessed on 22 June 2021).
78. Kang MS, Oh JY, Kwon YK, Lee DY, Jeong OM, Choi BK, et al. Public health significance of major genotypes of Salmonella enterica serovar Enteritidis present in both human and chicken isolates in Korea. Res Vet Sci. 2017;112:125–31. https://doi.org/10.1016/j.rvsc.2017.02.010.
79. Elez RMMA, Elsohaby I, El-Gazzar N, Tolba HMN, Abdelfatah EN, Abdellatif SS, et al. Antimicrobial resistance of salmonella enteritidis and salmonella typhimurium isolated from laying

hens, table eggs, and humans with respect to antimicrobial activity of biosynthesized silver nanoparticles. Animals. 2021;11(12):3554. https://doi.org/10.3390/ani11123554.

80. Siddique A, Azim S, Ali A, Andleeb S, Ahsan A, Imran M, et al. Antimicrobial resistance profiling of biofilm forming non typhoidal salmonella enterica isolates from poultry and its associated food products from Pakistan. Antibiotics. 2021;10(7):785. https://doi.org/10.3390/antibiotics10070785.
81. Wang W, Chen J, Shao X, Huang P, Zha J, Ye Y. Occurrence and antimicrobial resistance of Salmonella isolated from retail meats in Anhui, China. Food Sci Nutr. 2021;9(9):4701–10. https://doi.org/10.1002/fsn3.2266.
82. Thung TY, Mahyudin NA, Basri DF, Wan Mohamed Radzi CWJ, Nakaguchi Y, Nishibuchi M, et al. Prevalence and antibiotic resistance of Salmonella Enteritidis and Salmonella Typhimurium in raw chicken meat at retail markets in Malaysia. Poult Sci. 2016;95(8):1888–93. https://doi.org/10.3382/ps/pew144.
83. Dione MM, Ikumapayi UN, Saha D, Mohammed NI, Geerts S, Ieven M, et al. Clonal differences between non-typhoidal Salmonella (NTS) recovered from children and animals living in close contact in the Gambia. PLoS Negl Trop Dis. 2011;5(5):e1148. https://doi.org/10.1371/journal.pntd.0001148.
84. Barua H, Biswas PK, Talukder KA, Olsen KEP, Christensen JP. Poultry as a possible source of non-typhoidal salmonella enterica serovars in humans in Bangladesh.Vet Microbiol. 2014;168:372–80. https://doi.org/10.1016/j.vetmic.2013.11.020.
85. Sharma J, Kumar D, Hussain S, Pathak A, Shukla M, Prasanna Kumar V, et al. Prevalence, antimicrobial resistance and virulence genes characterization of nontyphoidal Salmonella isolated from retail chicken meat shops in Northern India. Food Control. 2019 Aug 1;102:104–11. https://doi.org/10.1016/j.foodcont.2019.01.021.
86. Gemeda BA, Amenu K, Magnusson U, Dohoo I, Hallenberg GS, Alemayehu G, et al. Antimicrobial Use in Extensive Smallholder Livestock Farming Systems in Ethiopia: Knowledge, Attitudes, and Practices of Livestock Keepers. Front Vet Sci. 2020;7(55):1–15. https://doi.org/10.3389/fvets.2020.00055.
87. Hosain MZ, Lutful Kabir SM, Kamal MM. Antimicrobial uses for livestock production in developing countries. Vet World. 2021;14(1):210–21. https://doi.org/10.14202/VETWORLD.2021.210-221.
88. Saravanan S, Purushothaman V, Murthy TRGK, Sukumar K, Srinivasan P, Gowthaman V, et al. Molecular Epidemiology of Nontyphoidal Salmonella in Poultry and Poultry Products in India: Implications for Human Health. Indian J Microbiol. 2015;55(3):319–26. https://doi.org/10.1007/s12088-015-0530-z.

89. Baran A, Erdoğan A, Kavaz A, Adigüzel MC. Some specific microbiological parameters and prevalence of salmonella spp. In retail chicken meat from Erzurum province, Turkey and characterization of antibiotic resistance of isolates. Bioscience Journal. 2019 ;35(3):878–91. https://doi.org/10.14393/BJ-v35n3a2019-42187.
90. Onyango D, Machoni F, Kakai R, Waindi EN. Multidrug resistance of Salmonella enterica serovars Typhi and Typhimurium isolated from clinical samples at two rural hospitals in Western Kenya. J Infect Dev Ctries. 2008;2(2):106–11. https://doi.org/10.3855/t2.2.106.
91. Yesigat T, Jemal M, Birhan W. Prevalence and Associated Risk Factors of Salmonella, Shigella, and Intestinal Parasites among Food Handlers in Motta Town, North West Ethiopia. Can J Infect Dis Med Microbiol. 2020;2020:6425946. https://doi.org/10.1155/2020/6425946.
92. Abd El-Aziz NK, Tartor YH, Gharieb RMA, Erfan AM, Khalifa E, Said MA, et al. Extensive Drug-Resistant Salmonella enterica Isolated From Poultry and Humans: Prevalence and Molecular Determinants Behind the Co-resistance to Ciprofloxacin and Tigecycline. Front Microbiol. 2021;12:738784. https://doi.org/10.3389/fmicb.2021.738784.
93. Archer EW, Chisnall T, Tano-Debrah K, Card RM, Duodu S, Kunadu APH. Prevalence and genomic characterization of Salmonella isolates from commercial chicken eggs retailed in traditional markets in Ghana. Front Microbiol. 2023;14:1283835. https://doi.org/10.3389/fmicb.2023.1283835.
94. Beyene T, Endalamaw D, Tolossa Y, Feyisa A. Evaluation of rational use of veterinary drugs especially antimicrobials and anthelmintics in Bishoftu, Central Ethiopia. BMC Res Notes.2015 Sep 28;8(482). https://doi.org/10.1186/s13104-015-1466-4.
95. 96. Guo K, Zhao Y, Cui L, Cao Z, Zhang F, Wang X, et al. The Influencing Factors of Bacterial Resistance Related to Livestock Farm: Sources and Mechanisms. Front Anim Sci. 2021;2:650347. https://doi.org/10.3389/fanim.2021.650347.
96. Samreen, Ahmad I, Malak HA, Abulreesh HH. Environmental antimicrobial resistance and its drivers: a potential threat to public health. J Glob Antimicrob Resist. 2021;27:101–11. https://doi.org/10.1016/j.jgar.2021.08.001.
97. Mir R, Salari S, Najimi M, Rashki A. Determination of frequency, multiple antibiotic resistance index and resistotype of Salmonella spp. in chicken meat collected from southeast of Iran. Vet Med Sci. 2022;8(1):229–36. https://doi.org/10.1002/vms3.647.

Click here to access/download
Supporting Information
S1 Figure.pdf
